# Clinical Comparator Dependence of Inflammatory and Hematologic Biomarker Discrimination for Sepsis: A Matched Retrospective Study Using MIMIC-IV Emergency Department Data

**DOI:** 10.3390/jcm15218489

**Published:** 2026-11-01

**Authors:** Salvador Sánchez-Vela, Jorge Martín Llaca-Díaz, Erik Alejandro Díaz-Chuc, Erik Alejandro San Miguel-Garay, Sergio Ayala-De la Cruz

**Affiliations:** Departamento de Patología Clínica, Facultad de Medicinay Hospital Universitario “Dr. José Eleuterio González”, Universidad Autónoma de Nuevo León, Monterrey 64460, Nuevo León, Mexico; salvador.sanchezv@uanl.edu.mx (S.S.-V.); jorge.llacadz@uanl.edu.mx (J.M.L.-D.); ediaz.me0085@uanl.edu.mx (E.A.D.-C.); esanmiguel2037528@uanl.edu.mx (E.A.S.M.-G.)

**Keywords:** sepsis, biomarkers, C-reactive protein, lymphocyte count, emergency service, hospital

## Abstract

**Background**: The diagnostic performance of sepsis biomarkers may vary according to the clinical comparator population. We evaluated 11 routinely available inflammatory and hematologic biomarkers in two matched diagnostic settings. **Methods**: We conducted a retrospective observational study using linked MIMIC-IV, MIMIC-IV-ED, and MIMIC-IV-Note data. Adult emergency department episodes were classified as sepsis, suspected infection without sepsis, or controls without suspected infection. Comparison-specific profile matching retained the same 525 sepsis episodes in both analyses, together with 214 controls without suspected infection and 315 episodes with suspected infection without sepsis. Biomarker discrimination was assessed using area under the receiver operating characteristic curve (AUROC) with DeLong inference. **Results**: Against controls without suspected infection, C-reactive protein (CRP) had the highest AUROC (0.786; 95% CI, 0.748–0.823), followed by absolute lymphocyte count (0.654; 0.613–0.696) and the neutrophil-to-lymphocyte ratio (NLR; 0.636; 0.594–0.677). Against suspected infection without sepsis, absolute lymphocyte count had the highest AUROC (0.677; 0.641–0.714), followed by NLR (0.626; 0.588–0.664), whereas CRP discrimination decreased to 0.555 (0.514–0.595). The cross-setting difference for CRP was 0.231 (95% CI, 0.183–0.278; Holm-adjusted *p* <0.001). Sensitivity analyses produced similar qualitative findings. **Conclusions**: Biomarker discrimination for sepsis varied according to the clinical comparator population, with the magnitude of cross-setting change differing across biomarkers. CRP showed the largest change, whereas absolute lymphocyte count retained modest discrimination in both settings. These findings emphasize the importance of interpreting biomarker performance within clinically relevant diagnostic contrasts.

## 1. Introduction

Sepsis is defined as life-threatening organ dysfunction caused by a dysregulated host response to infection and remains a major source of morbidity and mortality worldwide [1,2]. Distinguishing sepsis from infection without sepsis is particularly relevant during early acute care, when patients are evaluated before the subsequent clinical course is fully apparent. Recent emergency department (ED) studies have therefore examined biomarkers at or near presentation as potential tools for early clinical characterization [3].

Routinely available inflammatory and hematologic measurements are attractive in this setting because they are commonly obtained during the initial evaluation. C-reactive protein (CRP) has been extensively studied as an inflammatory marker in sepsis, while complete blood count parameters and derived indices (including leukocyte differential and ratios such as the neutrophil-to-lymphocyte ratio [NLR]) provide additional information on the host inflammatory response. However, their reported diagnostic performance has not been homogeneous. Recent systematic reviews have reported substantial between-study heterogeneity and variable pooled performance for sepsis biomarkers including CRP, while a recent ED cohort with suspected infection found only modest discrimination for several routinely available markers [4,5,6].

An important but incompletely addressed source of this variability is the clinical spectrum in which diagnostic performance is evaluated. Recent empirical evidence shows that diagnostic sensitivity and specificity vary with disease prevalence and participant spectrum, indicating that accuracy is population-dependent [7]. Studies that compare separately selected or clinically distant cases and controls may therefore estimate different contrasts and raise concerns about applicability to real diagnostic pathways [8]. This issue is particularly relevant to sepsis because patients without suspected infection and patients with suspected infection without sepsis represent distinct diagnostic challenges. Previous three-group studies have shown that inflammatory markers may separate infected from noninfected patients more readily than it will separate sepsis patients from those with an infection without sepsis [9]. Timing may further contribute to this variability; recent guidance emphasizes interpreting biomarkers in relation to sampling time and serial kinetics [10]. Estimates obtained at different stages of acute care may therefore not be directly interchangeable.

The extent to which the discrimination of routinely available inflammatory and hematologic biomarkers changes when the same sepsis cases are evaluated against clinically distinct comparator populations remains unclear. We therefore compared the discrimination of 11 routine inflammatory and hematologic biomarkers in two matched clinical settings, namely, sepsis versus controls without suspected infection and sepsis versus suspected infection without sepsis, retaining the same sepsis cohort across both comparisons. We hypothesized that biomarker discrimination would differ according to the clinical comparator population.

## 2. Materials and Methods

### 2.1. Study Design and Data Source

We conducted a retrospective observational study using linked data from MIMIC-IV version 3.1, MIMIC-IV-ED version 2.2, and MIMIC-IV-Note version 2.2 [11,12,13,14]. The source cohort comprised adult ED stays recorded in MIMIC-IV-ED at Beth Israel Deaconess Medical Center, Boston, Massachusetts, USA. MIMIC-IV-ED contains ED stays from 2011 through 2019; accordingly, this interval defined the source study period. Data for the present study were accessed in August 2025. This study was reported in accordance with the Strengthening the Reporting of Observational Studies in Epidemiology (STROBE) statement.

MIMIC-IV-ED stay information was linked to hospital, laboratory, microbiology, medication, diagnosis, derived data, and discharge note information from MIMIC-IV and MIMIC-IV-Note using the available patient-, hospitalization-, and stay-level identifiers together with an episode-level identifier constructed for the study. Temporal ascertainment of laboratory measurements, suspected infection, and organ dysfunction was anchored to ED arrival.

### 2.2. Cohort Construction and Laboratory Data Selection

The source cohort was restricted to ED episodes with at least one complete blood count (CBC) specimen obtained within 0–24 h of ED arrival. A specimen was considered complete for study purposes when valid platelet, absolute neutrophil, absolute lymphocyte, and absolute monocyte counts were available from the same specimen. The index CBC was defined as the earliest such complete specimen within the 0–24 h window.

CBC-derived ratios were calculated from the index specimen. The neutrophil-to-lymphocyte ratio (NLR), platelet-to-lymphocyte ratio (PLR), and monocyte-to-lymphocyte ratio (MLR) were calculated only when the lymphocyte count was greater than zero. The neutrophil-to-monocyte ratio (NMR) was calculated only when the monocyte count was greater than zero. Zero denominators were therefore retained as missing (not replaced by pseudo counts).

CRP measurements were standardized to mg/L and restricted to valid values obtained within 0–24 h of ED arrival. When more than one valid CRP measurement was available, the index CRP was selected according to its absolute temporal proximity to the index CBC. Eligibility for the primary analytical cohort required a valid index CRP and complete, finite NLR, PLR, MLR, and NMR values. When a patient had more than one otherwise-eligible ED episode, only the first eligible episode was retained. Participant selection is summarized in Figure 1.

### 2.3. Suspected Infection and Sepsis Classification

Suspected infection was identified from qualifying antimicrobial and microbiological events using a prespecified temporal pairing algorithm derived from the Sepsis-3 framework [15,16]. When antimicrobial therapy occurred first or at the same time as microbiological sampling, the microbiology event was required to occur within the subsequent 24 h. When microbiological sampling occurred first, antimicrobial therapy was required to begin within the subsequent 72 h. The earlier event in a qualifying pair defined the suspected infection onset, which was required to occur within 0–24 h of ED arrival.

When multiple qualifying pairs were present for the same episode, one index pair was selected deterministically. Selection prioritized the earliest suspected infection onset, followed by linkage of both events to the same hospitalization, the shortest temporal gap between events. Prespecified microbiology specimen types representing screening, serologic, postmortem, or otherwise nonqualifying specimens were excluded from the suspected-infection algorithm.

Sequential organ failure assessment (SOFA) components were derived from structured clinical data within the 0–24 h window after ED arrival [15,17]. A preliminary Sepsis-3 classification was generated from suspected infection status and the structured SOFA score. The phenotype used for the final manuscript analyses was subsequently revised using the clinical review procedure described below.

### 2.4. Biomarker Definitions

The eleven biomarkers evaluated were CRP, white blood cell count (WBC), absolute neutrophil count (ANC), absolute lymphocyte count (ALC), absolute monocyte count (AMC), NLR, PLR, MLR, NMR, the systemic inflammation response Index (SIRI) [18], and the systemic immune inflammation index (SII) [19].

The four CBC ratios were defined as NLR = neutrophil count/lymphocyte count, PLR = platelet count/lymphocyte count, MLR = monocyte count/lymphocyte count, and NMR = neutrophil count/monocyte count. The composite inflammatory indices were calculated as SIRI = neutrophil count × monocyte count/lymphocyte count and SII = neutrophil count × platelet count/lymphocyte count.

All CBC-derived biomarkers were calculated from the same index CBC specimen. CRP was obtained from the index CRP measurement defined above. For ROC analyses, lower ALCs were treated as indicating greater probability of sepsis, whereas higher values indicated greater probability of sepsis for the rest of biomarkers.

### 2.5. Clinical Review, Exclusion Criteria, Revised Phenotype Classification, and Anatomical Site Validation

Clinical review was performed by a physician reviewer with training in clinical pathology and was organized as three separate review tasks: confirmation of potential clinical exclusion criteria, anatomical site adjudication, and manual review of flagged SOFA components. These tasks were conducted separately, with approximately 2–3 weeks between completion of one task and initiation of the next. During review, the reviewer was not provided with the final study phenotype or structured biomarker results.

Potential clinical exclusion criteria were first identified algorithmically from diagnosis, medication, and other structured clinical data. Flagged episodes were then manually reviewed to confirm whether a prespecified exclusion criterion was present. The grouped exclusion categories used for reporting were immunomodulatory treatment, hematologic disease or treatment capable of altering hematopoiesis or platelet measurements, active malignancy or antineoplastic treatment, and active pregnancy. Individual exclusion criteria were not mutually exclusive. Anatomical site adjudication was performed for all episodes with suspected infection, including episodes subsequently classified as sepsis. The reviewer used clinical documentation within the prespecified temporal window and assigned a broad anatomical site according to a predefined site dictionary while blinded to the final phenotype classification and structured biomarker results.

Manual SOFA review was restricted to components flagged as lacking usable structured data. Structured SOFA component scores, when available, were retained. For flagged components, adjudication was based on clinical documentation within the prespecified 0–24 h window after ED arrival (information outside this window was not considered). A component score was assigned when the note explicitly documented the corresponding SOFA score or when sufficient information was available to derive the score (such as PaO_2_ and FiO_2_ for the respiratory component) within the time window. When neither structured nor sufficient manually reviewable information was available, the component remained unavailable and contributed no additional points to the observed revised SOFA sum.

Final phenotype classification was algorithmic (not reviewer assigned). Episodes with suspected infection and a revised observed SOFA total ≥2 were classified as sepsis. Episodes with suspected infection and a revised observed SOFA total <2 were classified as suspected infection without sepsis. Episodes without suspected infection were classified as controls without suspected infection.

Reproducibility of anatomical site classification was evaluated in a separate validation sample of 200 episodes selected in proportion to the frequency of the broad anatomical site categories. A second physician reviewer independently assigned the anatomical site using the same predefined criteria, while blinded to the original classification, study phenotype, structured biomarker results, and anatomical site frequencies. Agreement was assessed before category collapse using overall exact agreement and unweighted Cohen’s κ, with 95% confidence intervals estimated from 5000 case-level nonparametric bootstrap replicates. Acceptable reproducibility was defined a priori as κ ≥0.60 [20].

### 2.6. Missing Data, Study Size, and Bias Reduction Measures

Missing values for biomarker measurements required for analytical cohort eligibility were not imputed. Episodes lacking a valid CRP value within the primary window or complete and finite NLR, PLR, MLR, and NMR values were excluded before analysis. The treatment of unavailable SOFA components differed from this complete case biomarker requirement and followed the structured/manual fallback procedure described in Section 2.5. Matching model frames were required to contain all matching covariates. No a priori sample size or power calculation was performed. Study size was determined by the number of episodes satisfying the prespecified eligibility and clinical exclusion criteria in the source cohort and, for the primary analyses, by the subsets retained through profile matching. Several design measures were used to reduce potential bias. Only the first eligible episode per patient was retained to avoid repeated observations from the same individual. Matching covariates and balance thresholds were specified before the data analysis. Unmatched and coagulation sensitivity analyses were performed to assess the stability of the primary findings.

### 2.7. Matching

The two comparison settings were constructed independently using optimization-based profile matching implemented with the MatchIt package [21,22,23]. The sepsis group was specified as the target profile and retained intact, whereas the largest subset of comparator episodes satisfying the prespecified covariate balance constraints was selected. The MatchIt average treatment effect on the treated (ATT) specification was used operationally to preserve the sepsis group as the target population during subset selection. No causal treatment effect was estimated. Matching covariates were prespecified before the biomarker discrimination analyses based on clinical relevance and recent evidence relating demographic and sociodemographic characteristics to variation in inflammatory and hematologic profiles [24,25,26]. Age, sex, race, and insurance status were included as baseline demographic and sociodemographic matching variables. Chronic pulmonary disease, chronic liver disease, chronic renal disease, and diabetes were also prespecified because recent evidence has associated these conditions with alterations in systemic inflammatory or hematologic indices relevant to the biomarkers evaluated in this study [27,28,29,30]. These baseline variables were used in both matching strategies to reduce demographic and comorbidity differences that could influence biomarker distributions. This common framework was intended to support a more clinically demanding evaluation of discrimination between groups with comparable measured baseline profiles.

For the sepsis versus controls without suspected infection (SvC) comparison, these baseline variables were supplemented by observed noncoagulation SOFA category and the number of unavailable noncoagulation SOFA components to improve comparability with respect to nonhematologic organ dysfunction. For the sepsis versus suspected infection without sepsis (SvI) comparison, the same baseline variables were supplemented by suspected infection site category to improve clinical comparability within the suspected infection population.

Balance constraints were set at 0.10 standardized units for all matching covariates. Covariate balance was evaluated using absolute standardized mean differences (SMDs), with an absolute SMD <0.10 prespecified as an operational criterion for acceptable balance [21,23].

### 2.8. Primary Discrimination Analyses

Biomarker discrimination was evaluated separately in the two profile-matched comparison settings. For each of the 11 biomarkers, the empirical area under the receiver operating characteristic curve (AUROC) and its 95% confidence interval were estimated using the nonparametric DeLong method [31]. Within each comparison setting, all pairwise differences between biomarker AUROCs were evaluated using paired DeLong tests. With 11 biomarkers, this yielded 55 pairwise comparisons per setting. To control the family-wise error rate, *p* values were adjusted using the Holm procedure [32] separately within the 55 comparisons from each setting. All tests were two-sided; statistical significance was defined as a *p* value < 0.05.

### 2.9. Cross-Setting Comparison of Discrimination

To formally evaluate whether biomarker discrimination differed between the two clinical comparison settings, the cross-setting difference for each biomarker was defined as the AUROC for sepsis versus controls without suspected infection minus the AUROC for sepsis versus suspected infection without sepsis. Because the same sepsis episodes contributed to both matched comparisons whereas the comparator samples were distinct, the two AUROC estimates were statistically dependent. This dependence was accounted for using a nonparametric structural-component approach based on the DeLong framework for correlated ROC curves with incomplete overlap [31,33]. The estimated covariance was incorporated into the variance used to obtain 95% confidence intervals and two-sided Wald tests for the cross-setting AUROC differences. Inference was performed on the matched samples obtained in the primary analysis, without repeating the matching process. *p* values for the 11 biomarker-specific cross-setting comparisons were adjusted using the Holm procedure.

### 2.10. Sensitivity Analyses

Two sensitivity analyses were performed in the clinically reviewed cohort to assess the stability of the primary findings. First, biomarker discrimination was reevaluated in the unmatched study populations, without restricting the analyses to comparator episodes retained through profile matching. AUROC and pairwise biomarker comparisons were recalculated using the same statistical procedures and setting specific multiplicity adjustment as in the primary analyses. Second, a coagulation sensitivity analysis was performed to assess whether the findings were influenced by sepsis classifications that depended on platelet related points in the coagulation component of SOFA. Sepsis episodes for which meeting the SOFA ≥2 classification threshold depended on points contributed by the coagulation component were excluded before rematching. For the SvC comparison, the control candidate pool was subsequently restricted to noncoagulation SOFA categories represented among the remaining eligible sepsis episodes to ensure common support for this matching variable. This restriction did not remove eligible sepsis episodes. The comparator candidate pool for the SvI comparison was otherwise retained according to the original specification. Profile matching was then repeated using the original comparison-specific covariates and balance constraints, and biomarker AUROC and pairwise comparisons were recalculated using the same procedures as in the primary analyses.

A post hoc sensitivity analysis evaluated the ten CBC biomarkers without requiring CRP. Each patient contributed the first episode with valid CRP within 0–24 h and four complete, finite CBC ratios or, if none qualified, the first CBC episode with complete, finite ratios. Automated phenotypes used suspected infection status and structured SOFA, with unavailable components adding no points. No clinical review, adjudication, exclusions, or matching were applied. Characteristics were summarized by CRP eligibility using descriptive statistics and absolute SMDs (Supplementary Table S7). AUROC estimates with DeLong 95% CIs were estimated within each group for both diagnostic comparisons, using the directions specified in Section 2.4 (Supplementary Table S8).

### 2.11. Post Hoc Analysis of NLR Components

One post hoc analysis was conducted after inspection of the primary biomarker discrimination results. Because of the relative performance of NLR and ALC, this analysis examined the relative statistical contributions of the neutrophil and lymphocyte components of NLR in the primary matched comparison samples. Detailed methods are provided in the Supplementary Methods. Nonlinear associations were modeled using spline functions in accordance with epidemiologic guidance on spline application and interpretation [34].

### 2.12. Statistical Software

Data processing and statistical analyses were performed using R version 4.4.3. Data manipulation and database operations included use of DuckDB version 1.5.0. ROC analyses, including AUROC estimation, DeLong confidence intervals, and paired DeLong comparisons, were implemented using the pROC package version 1.19.0.1 [35]. Profile matching was implemented using MatchIt version 4.7.2 [22], with covariate balance diagnostics obtained using cobalt version 4.6.3. Optimization was performed using HiGHS through the highs R package version 1.12.0-3 [36].

### 2.13. Ethics and Data Access

The study protocol was reviewed and approved by the Ethics and Research Committee of the Faculty of Medicine, Universidad Autónoma de Nuevo León (approval #PC-250004), which granted a waiver of informed consent for this secondary analysis of deidentified data. The MIMIC research resources contain deidentified health data. The original collection and sharing of MIMIC data were reviewed by the Institutional Review Board of Beth Israel Deaconess Medical Center, with a waiver of informed consent for the data sharing resource. Access to MIMIC-IV, MIMIC-IV-ED, and MIMIC-IV-Note was obtained through PhysioNet under the applicable credentialing, training, and Data Use Agreement requirements.

## 3. Results

### 3.1. Participant Flow, Matched Samples, and Anatomical Site Validation

Of 52,994 episodes in the CBC eligible phenotype cohort, 4316 had a valid CRP measurement within 0–24 h of ED arrival. After criteria requiring complete and finite NLR, PLR, MLR, and NMR values were imposed, 4289 episodes remained. Restriction to the first eligible episode per patient yielded 3889 episodes, of which 167 were excluded because no usable discharge note was available for revised clinical classification. A further 513 episodes met one or more prespecified clinical exclusion criteria, resulting in a final analytical cohort of 3209 episodes (Figure 1). The analytical cohort comprised 525 episodes with sepsis, 1497 controls without suspected infection, and 1187 episodes with suspected infection without sepsis. Profile matching retained all 525 sepsis episodes in both comparisons, together with 214 controls without suspected infection and 315 episodes with suspected infection without sepsis.

Anatomical site classification met the prespecified reproducibility criterion, with an unweighted Cohen’s κ of 0.783 (95% bootstrap CI, 0.717–0.843). Overall exact agreement was 81.5% (95% bootstrap CI, 76.0–86.5%). Among the 37 discordant classifications, the most frequent transitions were gastrointestinal/intraabdominal/hepatobiliary infection to unknown site (6/37, 16.2%), bone/joint to skin/soft tissue infection (5/37, 13.5%), and skin/soft tissue to device/procedure-related infection (3/37, 8.1%). Together, these three transitions accounted for 37.8% of all disagreements.

### 3.2. Matching and Covariate Balance

Profile matching retained all 525 sepsis episodes in both comparison settings (Table 1). Among the 1497 available controls without suspected infection, 214 were retained (total of 739 episodes). Among the 1187 episodes with suspected infection without sepsis, 315 were retained (total of 840 episodes).

Substantial covariate imbalance was present before matching, with maximum absolute standardized mean differences of 2.58 in the SvC comparison and 0.619 in the SvI comparison. After profile matching, all modeled covariates satisfied the prespecified absolute SMD <0.10 balance criterion in both comparisons. Detailed balance diagnostics are shown in Supplementary Figure S1. Additional characteristics of the matched samples are summarized in Supplementary Tables S1 and S2.

Following structured data extraction and targeted manual review, respiratory SOFA component data were available in 30/525 sepsis episodes (5.7%), 7/214 controls without suspected infection (3.3%), and 0/315 episodes with suspected infection without sepsis (0%). Neurologic component data were available in 208/525 (39.6%), 71/214 (33.2%), and 22/315 (7.0%), while hepatic component data were available in 413/525 (78.7%), 178/214 (83.2%), and 181/315 (57.5%). Coagulation component data were available in all episodes, and cardiovascular and renal component data were available in at least 99.5% of episodes in each group. Component scores and data availability are detailed in Supplementary Table S1.

### 3.3. Primary Biomarker Discrimination

In the matched SvC comparison (Figure 2 and Table 2), CRP showed the highest discrimination, with an AUROC of 0.786 (95% CI, 0.748–0.823). ALC ranked second (AUROC, 0.654; 95% CI, 0.613–0.696), followed by NLR (0.636; 95% CI, 0.594–0.677) and MLR (0.630; 95% CI, 0.587–0.672). The remaining hematologic indices showed lower discrimination, with AUROC ranging from 0.497 for AMC to 0.605 for SIRI. CRP significantly outperformed each of the other 10 biomarkers in this comparison after adjustment. The smallest difference was observed relative to ALC (ΔAUROC, 0.131; 95% CI, 0.080–0.183; Holm-adjusted *p* < 0.001), whereas the largest was relative to AMC (ΔAUROC, 0.289; 95% CI, 0.238–0.340; Holm-adjusted *p* < 0.001). Overall, 27 of 55 pairwise biomarker comparisons remained significant after Holm adjustment.

In the SvI comparison (Figure 2 and Table 2), the pattern of relative biomarker discrimination differed. ALC had the highest AUROC (0.677; 95% CI, 0.641–0.714), followed by NLR (0.626; 95% CI, 0.588–0.664) and MLR (0.591; 95% CI, 0.552–0.630). CRP ranked sixth, with an AUROC of 0.555 (95% CI, 0.514–0.595). ALC discriminated significantly better than CRP (ΔAUROC for CRP minus lymphocyte count, −0.122; 95% CI, −0.172 to −0.073; Holm-adjusted *p* < 0.001), and NLR also outperformed CRP (ΔAUROC, −0.071; 95% CI, −0.113 to −0.030; Holm-adjusted *p* = 0.021). CRP performed significantly better than AMC (ΔAUROC, 0.127; 95% CI, 0.078–0.176; Holm-adjusted *p* < 0.001), whereas its differences from MLR, NMR, SIRI, PLR, SII, WBC, and ANC did not remain significant after multiplicity adjustment. Overall, 35 of 55 pairwise comparisons were significant after Holm adjustment. The complete pairwise AUROC comparisons for both matched settings are shown in Supplementary Figure S2.

### 3.4. Cross-Setting Differences in Biomarker Discrimination

Cross-setting differences in discrimination were heterogeneous across biomarkers (Table 3). The largest difference was observed for CRP, whose AUROC decreased from 0.786 to 0.555, corresponding to a ΔAUROC of 0.231 (95% CI, 0.183–0.278; Holm-adjusted *p* < 0.001). After Holm adjustment across the 11 biomarkers, significant cross-setting differences were also observed for PLR (ΔAUROC, 0.072; 95% CI, 0.029–0.114; adjusted *p* = 0.010), AMC (0.069; 95% CI, 0.026–0.112; adjusted *p* = 0.014), and SII (0.066; 95% CI, 0.023–0.108; adjusted *p* = 0.021). Differences for the remaining seven biomarkers did not remain significant after Holm adjustment.

Notably, the discrimination of ALC, NLR, MLR, and NMR was comparatively stable between the two settings. The corresponding cross-setting differences were −0.023 for ALC, 0.010 for NLR, 0.038 for MLR, and −0.012 for NMR, none of which were statistically significant after Holm adjustment (Table 3).

### 3.5. Sensitivity Analyses

#### 3.5.1. Unmatched Analyses

In the unmatched populations, discrimination estimates were generally higher than in the matched analysis for the comparison between sepsis and controls without suspected infection. The largest increases were observed for NLR (AUROC 0.636 to 0.725), ALC (0.654 to 0.742), MLR (0.630 to 0.715), and SIRI (0.605 to 0.684). CRP remained the highest performing biomarker, with its AUROC increasing modestly from 0.786 to 0.810. Differences between matched and unmatched estimates were smaller in the SvI comparison. The largest absolute change was observed for NMR (AUROC 0.579 to 0.545; Δ = −0.034), followed by MLR (0.591 to 0.625; Δ = 0.034). NLR was essentially unchanged (0.626 versus 0.629), while CRP increased from 0.555 to 0.570. Complete unmatched AUROC estimates and pairwise biomarker comparisons are provided in Supplementary Table S3 and Supplementary Figure S3, respectively.

#### 3.5.2. Coagulation Sensitivity Analysis

Of the 525 sepsis episodes in the primary cohort, 101 (19.2%) were classified as coagulation dependent and were excluded in the coagulation sensitivity analysis, leaving 424 sepsis episodes. After the common support restriction and rematching, the SvC comparison included 424 sepsis episodes and 166 controls, whereas the SvI comparison included 424 sepsis episodes and 270 comparator episodes. All matching covariates again satisfied the prespecified balance criterion. Biomarker discrimination remained broadly similar to the primary matched analysis. In the SvC comparison, the largest absolute AUROC change was 0.035 for ALC, followed by 0.033 for SII and 0.032 for CRP. NLR was essentially unchanged (ΔAUROC, −0.001). In the SvI comparison, all absolute AUROC changes were ≤0.026, with CRP changing from 0.555 to 0.561 and NLR from 0.626 to 0.630. Complete AUROC estimates and pairwise biomarker comparisons from the coagulation sensitivity analysis are shown in Supplementary Table S4 and Supplementary Figure S4, respectively.

#### 3.5.3. Post Hoc Sensitivity Analysis by CRP Eligibility

The post hoc sensitivity analysis included 38,089 episodes (one episode per patient): 3889 in the CRP-eligible group and 34,200 in the CBC-only group. The CRP-eligible group corresponded to the 3889 episodes shown in Figure 1 before clinical review and exclusions. Compared with the CBC-only group, patients in the CRP-eligible group were younger (median age, 59 versus 62 years), more frequently had an ED disposition of admission (95.1% versus 86.0%), and more frequently met the automated suspected infection criteria (51.8% versus 40.7%). Clinical and laboratory characteristics are summarized in Supplementary Table S7.

ALC and NLR yielded the two highest AUROC point estimates among the CBC biomarkers in both groups and both diagnostic comparisons. In the SvC comparison, the AUROC estimates for ALC were 0.737 and 0.725 in the CRP-eligible and CBC-only groups, respectively. The AUROC estimates for NLR were 0.722 and 0.717. In the SvI comparison, AUROC were 0.687 and 0.653 for ALC and 0.628 and 0.596 for NLR, respectively. Complete AUROC estimates and 95% confidence intervals are provided in Supplementary Table S8.

### 3.6. Post Hoc Analysis of NLR Components

Because NLR did not outperform ALC in either matched comparison, we conducted a post hoc component analysis to examine the relative contributions of absolute lymphocyte and neutrophil counts (Supplementary Table S6 and Supplementary Figure S5). In the primary spline specification with three degrees of freedom per component, lymphocyte count was associated with a substantial improvement in model fit after accounting for neutrophil count in both matched comparison settings (Holm-adjusted *p* < 0.001 for both). The corresponding increases in apparent AUROC were 0.109 for sepsis versus controls without suspected infection and 0.130 for sepsis versus suspected infection without sepsis. The additional contribution of neutrophil count after accounting for lymphocyte count was smaller. Although model fit improved in both comparisons (Holm-adjusted *p* < 0.001 and *p* = 0.042, respectively), the corresponding increases in apparent AUROC were only 0.027 and 0.011. In the sensitivity specification with four degrees of freedom per component, the additional contribution of neutrophil count remained statistically significant for sepsis versus controls without suspected infection (Holm-adjusted *p* < 0.001) but was no longer statistically significant in the comparison of sepsis versus suspected infection without sepsis (Holm-adjusted *p* = 0.059). In contrast, the additional contribution of lymphocyte count remained statistically significant in both comparisons (Holm-adjusted *p* < 0.001 for both).

## 4. Discussion

In this retrospective matched analysis of ED episodes, biomarker discrimination varied across the two clinical comparator populations, but the magnitude of this variation was biomarker-specific. CRP showed by far the largest cross-setting change, decreasing from an AUROC of 0.786 when sepsis was compared with controls without suspected infection to 0.555 when the comparator consisted of patients with suspected infection without sepsis. Significant cross-setting differences after multiplicity adjustment were also observed for PLR, AMC, and SII, whereas differences for the remaining seven biomarkers did not remain significant. Relative biomarker performance also changed across the two diagnostic contrasts: CRP was the strongest discriminator against controls without suspected infection, whereas ALC and NLR both discriminated significantly better than CRP in the suspected infection comparison. ALC itself showed modest discrimination in both settings (AUROC 0.654 and 0.677, respectively), and NLR changed comparatively little. The SvI comparison is particularly relevant to the clinical assessment of patients with suspected infection, and discrimination in this setting was modest for all biomarkers. The observed AUROCs characterize discrimination in the evaluated samples and do not, by themselves, demonstrate clinical utility.

This pattern is consistent with recent evidence that diagnostic accuracy varies across patient spectra. Diagnostic discrimination is conditional on the distributions of test values among the cases and non-cases included in a particular evaluation and therefore should not be interpreted as an immutable, context-free property of a biomarker. Disease prevalence and participant spectrum are empirically associated with sensitivity and specificity across diagnostic accuracy studies [7]. Studies that compare clearly diseased cases with separately selected or clinically distant controls may therefore estimate a different diagnostic contrast from studies performed within more overlapping clinical populations, with implications for both risk of bias and applicability [8]. This distinction is important because variation in performance across patient spectra is not automatically evidence of bias. In the absence of evidence that one sampled population provides an inappropriate estimate for its intended clinical use, the more precise interpretation is that different populations pose different diagnostic discrimination problems.

The two comparator populations in the present study were deliberately constructed to address different clinical contrasts and were therefore not matched identically. For sepsis versus controls without suspected infection, the main comparability problem was avoiding an overly simple contrast between sepsis and otherwise low acuity controls. The common baseline covariates were therefore supplemented with observed noncoagulation SOFA category and the number of unavailable noncoagulation SOFA components. The retained controls should not be interpreted as healthy controls, as their median total observed SOFA score was 3 [IQR 2–4], and their median observed noncoagulation SOFA score was 2 [2,3,4] (Supplementary Table S1). For sepsis versus suspected infection without sepsis, the clinical comparability problem was different. Both groups met the same suspected infection framework, while observed organ dysfunction contributed directly to their classification as sepsis or non-septic suspected infection. The matching incorporated the adjudicated anatomical site of suspected infection, with the aim of reducing differences in infection source composition that could contribute to biomarker distributions independently of sepsis classification. Thus, the comparison-specific matching strategies were intended to address distinct sources of clinical noncomparability.

Keeping the sepsis group fixed provides a common case spectrum across the two comparisons. The same 525 sepsis episodes and their biomarker values contributed to both primary analyses. This makes changes associated with the comparator side more directly interpretable than when comparing entirely separate case-control samples. However, both the comparator populations and the variables used for matching differed between settings. The observed cross-setting AUROC differences therefore reflect the two comparison-specific matched settings and cannot be attributed exclusively to the comparator population. Matching cannot remove unmeasured clinical differences.

The unmatched sensitivity analysis further reduces concern that the principal contrast was created solely by the use of different matching strategies. Before matching, CRP showed an AUROC of 0.810 against controls without suspected infection and 0.570 against suspected infection without sepsis, preserving a marked difference similar to that seen in the primary matched analyses (Supplementary Table S3). ALC and NLR also retained higher AUROC than CRP in the unmatched suspected infection comparison. Matching therefore affected the magnitude of discrimination, as expected when the compared populations are restricted to improve covariate balance, but it did not generate a different CRP pattern.

CRP provides the clearest empirical illustration of comparator-associated discrimination in this study. Its AUROC decreased by 0.231 when the comparator shifted from controls without suspected infection to patients with suspected infection without sepsis. This finding aligns with recent work showing that CRP accuracy varies across clinical spectra. In a three-group study, Zincircioğlu et al. found that CRP differed between sepsis and patients without infection but did not clearly distinguish sepsis from infection without sepsis [9]. A recent meta-analysis that compared monocyte distribution width, procalcitonin, and CRP estimated a CRP summary ROC area of 0.86 but documented substantial heterogeneity across the contributing studies [5]. Conversely, a recent ED cohort restricted to patients with suspected infection found only modest discrimination for routine hematologic markers [6]. Cross-study differences cannot be attributed to comparator composition alone because setting, disease severity, timing, selection criteria, and phenotype definitions also vary. However, considered together with the within-study contrast observed here, they reinforce the importance of specifying what clinical distinction a reported biomarker AUROC represents.

The attenuation of CRP discrimination in the suspected infection comparison is also biologically plausible, but its mechanism cannot be established from the present data. The findings are consistent with substantial overlap in CRP-related inflammatory responses between sepsis and non-septic infection, which may contribute to the reduced separation observed when suspected infection is present on both sides of the comparison [9]. The study did not measure the pathways responsible for CRP elevation, and reduced discrimination in this setting does not imply that CRP lacks clinical relevance for other purposes.

ALC showed a different pattern. Its discrimination was modest but changed little across the two matched settings, and its AUROC of 0.677 against suspected infection without sepsis closely resembles the AUROC of 0.67 reported by Cancella De Abreu et al. [6] in a cohort of 1155 ED patients with suspected infection. This concordance supports the presence of a reproducible, although limited, discriminatory signal for ALC within clinically overlapping suspected infection populations. It does not establish ALC as a clinically useful standalone diagnostic test, nor does it demonstrate that ALC is intrinsically resistant to changes in comparator spectrum. The present study shows that its discrimination was relatively preserved across the two specific comparator populations evaluated here, in contrast to the marked change observed for CRP.

NLR similarly did not demonstrate a clear discriminatory advantage over ALC in either primary matched comparison. Because NLR is mathematically constructed from ANC and ALC, this observation motivated a post hoc analysis. In spline models, adding ALC after accounting for ANC count produced substantially larger improvements in model fit and apparent discrimination than adding ANC after accounting for ALC, particularly in the suspected infection comparison (Supplementary Table S6 and Supplementary Figure S5). These results are hypothesis generating and should not be interpreted as demonstrating that NLR is biologically “driven” by ALC or that ANC contributes no additional information. Rather, they illustrate that a derived index should demonstrate incremental information beyond the variables from which it is constructed rather than being assumed to be superior because of its mathematical manipulation [37].

The same caution applies to the other derived hematologic indices. PLR, MLR, NMR, SIRI, and SII did not show a consistent discriminatory advantage over simpler measurements across both clinical settings. This should not be interpreted as evidence that complex indices are inherently inferior. Several of these indices have primarily been studied for prognosis or mortality in established sepsis, which addresses a different clinical question from cross-sectional diagnostic discrimination [38,39,40,41]. Schupp et al. found that NLR poorly distinguished sepsis from septic shock and poorly predicted 30-day mortality, although it better discriminated blood-culture positivity within septic shock [42]. These results are not directly comparable with our cross-sectional contrasts, but they reinforce that the apparent utility of a derived index depends on the target population and endpoint. The conclusion supported by the present data is that greater mathematical complexity did not guarantee greater discrimination.

A further implication is that comparative biomarker performance may itself be spectrum dependent. In the first matched setting, CRP significantly outperformed all other evaluated biomarkers. In the suspected infection setting, ALC and NLR both significantly outperformed CRP, while several other pairwise differences narrowed. This does not imply that biomarker rankings necessarily reverse whenever the comparator changes. It does show that statements identifying a “best performing” biomarker require specification of the diagnostic population and task. Different biomarkers may be affected to different degrees when the non-target-condition population changes.

This point also connects the study to the broader problem of transportability. Methodological work on external validation has shown that observed discrimination can vary as case mix changes, even when changes in discrimination do not necessarily imply that the underlying predictor relationships have failed [43]. Large collections of external validations have likewise documented substantial variation in AUROC across clinical settings [44]. Although much of this methodological literature concerns multivariable prediction models rather than individual laboratory biomarkers, the general principle is relevant, a discrimination estimate is conditional on the population in which it is measured. The present findings provide a within study illustration of this problem for routine sepsis biomarkers. Performance estimated in one comparator spectrum should therefore not be assumed to transport unchanged to a clinically distinct diagnostic contrast.

Comparator composition may also contribute to the substantial heterogeneity observed across the sepsis biomarker literature. Recent syntheses involving CRP have reported considerable between-study heterogeneity, while individual studies vary in setting, sepsis definition, biomarker timing, assay methods, disease severity, and the composition of the non-sepsis population [4,5]. The present study cannot quantify how much of that heterogeneity is attributable to comparator spectrum. Recent diagnostic tests meta-analysis methodology emphasizes explicit investigation of clinically relevant sources of heterogeneity [45]. A generic label such as “non-sepsis” may therefore hide important differences if it combines patients without suspected infection, patients with suspected or confirmed infection without sepsis, critically ill non-septic patients, and other acute illnesses.

The coagulation focused sensitivity analysis provides additional support for the robustness of the main patterns. Excluding the 101 sepsis episodes whose classification depended on platelet related SOFA points and rematching the remaining cases preserved the qualitative findings: CRP remained substantially more discriminative against controls without suspected infection than against suspected infection without sepsis, whereas cross-setting changes for several other biomarkers remained smaller (Supplementary Table S4). This reduces concern that the principal findings were driven primarily by dependence of sepsis classification on platelet related SOFA scoring. It does not eliminate phenotype misclassification, residual confounding, or other dependencies introduced by the retrospectively reconstructed classification.

The post hoc sensitivity analysis provided additional context for the CRP eligibility requirement. The CRP-eligible and CBC-only groups differed in age, ED disposition, and the frequency of suspected infection (Supplementary Table S7). ALC and NLR had the highest AUROC estimates among the CBC biomarkers in both groups, although their SvI estimates were lower in the CBC-only group (Supplementary Table S8). However, this analysis used automated phenotype classification without the clinical adjudication, manual SOFA revision, and clinical exclusions applied in the primary analysis. This analysis cannot establish that the primary AUROC estimates were unaffected by the CRP restricted selection.

Microbiologic findings provide additional context for the clinical overlap within the suspected infection comparison. A valid reported microbiologic finding was identified in 40.2% of sepsis episodes and 37.1% of episodes with suspected infection without sepsis, while valid positive blood cultures were observed in 17.5% and 10.8%, respectively (Supplementary Table S5). These findings reinforce that the study phenotype represents suspected infection combined with observed organ dysfunction rather than microbiologically confirmed sepsis. Microbiology was not an independent reference standard, the absence of a positive finding does not exclude clinically important infection, and detection of an organism does not necessarily establish that it caused the acute syndrome. The microbiologic findings therefore further characterize the clinical overlap within the suspected infection comparison without providing an independent etiologic adjudication of infection.

The present study addresses diagnostic discrimination and not clinical utility. A higher AUROC indicates greater separation between the groups being compared, but it does not by itself establish that using the biomarker improves clinical decisions or patient outcomes. Evaluation of added diagnostic value requires determining whether a marker contributes information beyond what is already available from clinical assessment or other tests [37]. Clinical usefulness additionally depends on the consequences of false-positive and false-negative decisions and on clinically relevant decision thresholds [46]. Accordingly, the relatively high CRP AUROC observed in the first setting should not be interpreted as demonstrating clinical usefulness, just as an AUROC in the 0.60–0.70 range for lymphocyte count or NLR does not by itself establish either usefulness or irrelevance. Prospective evaluation, external validation across clinically relevant spectra, and assessment of incremental value would be required before translating these findings into clinical decision making.

Several limitations should be considered. First, this was a retrospective analysis of a single-center electronic health record database, and the results may not generalize to institutions with different patient populations, laboratory practices, infection-testing strategies, or thresholds for ordering inflammatory biomarkers. CRP availability was particularly selective, as only 4316 of 52,994 episodes meeting the CBC eligibility criteria had a valid CRP measurement within the prespecified window. The decision to order CRP may reflect clinical concern, diagnostic uncertainty, or the extent of investigation. Requiring its availability may therefore have selected patients whose clinical presentations and evaluation differed from those of the broader CBC-eligible population, potentially affecting discrimination estimates and limiting their applicability to episodes without eligible CRP measurements. Second, sepsis and suspected infection phenotypes were reconstructed retrospectively. Revised SOFA scoring retained usable structured component scores and incorporated information recovered through targeted manual review. Components that remained unavailable after this process contributed no additional points to the observed score. Residual missingness was particularly pronounced for the respiratory and neurologic components, and component availability differed across the matched groups (Supplementary Table S1). This may have led to underestimation of organ dysfunction and misclassification of some episodes with suspected infection as infection without sepsis. The direction and magnitude of any resulting bias in AUROC estimates remains uncertain.

Third, the comparator populations and variables used for matching differed between the two primary analyses and the observed cross-setting AUROC differences cannot be attributed exclusively to the comparator population. The persistence of the principal CRP pattern in the unmatched analysis reduces concern that it resulted only from the matching procedures. Nevertheless, matching addresses only measured variables, and residual differences in clinical presentation, infection source, treatment before biomarker measurement, illness timing, and other unmeasured factors may still have influenced the observed biomarker estimates.

Fourth, biomarkers were measured within the first 24 h after ED presentation rather than at a single uniform time point. This reflects real world data availability but introduces temporal heterogeneity and limits inference about performance at a specific moment of presentation. Fifth, the NLR component analysis was explicitly post hoc. Its apparent AUROCs were calculated in the same samples used for model fitting, and the observed asymmetry between lymphocyte and neutrophil contributions requires independent confirmation. Finally, the study evaluated individual biomarkers and mathematically derived indices rather than multivariable diagnostic models that integrate laboratory findings with clinical information. The results therefore characterize discrimination under different comparator spectra but do not establish whether any of these measurements would add clinically meaningful information.

## 5. Conclusions

The discrimination of routine inflammatory and hematologic biomarkers for sepsis differed across the two comparison-specific matched settings, and the magnitude of this variation differed across biomarkers. CRP provided the clearest example, showing substantially less separation when the comparator consisted of patients with suspected infection without sepsis, whereas ALC retained modest discrimination across the two settings examined. The same fixed sepsis cohort demonstrated that changes in the non-sepsis side of a diagnostic contrast can be accompanied by substantial changes in apparent and relative biomarker performance, without implying that all biomarkers respond in the same way. However, these findings should be interpreted with caution. Both matching strategies achieved balance on shared baseline covariates and incorporated additional variables relevant to each clinical contrast, with the aim of evaluating discrimination between more clinically comparable groups. Nevertheless, the additional matching covariates differed between settings, so the observed differences cannot be attributed exclusively to the comparator population. In the clinically relevant comparison between sepsis and suspected infection without sepsis, discrimination was modest for all biomarkers, and the observed AUROCs do not, by themselves, establish clinical utility. More broadly, these findings emphasize that diagnostic discrimination should be interpreted in relation to the clinically relevant population and diagnostic contrast in which it was estimated. Performance observed in one comparator spectrum should not be assumed to represent performance in another clinically distinct setting.

## Figures and Tables

**Figure 1:**
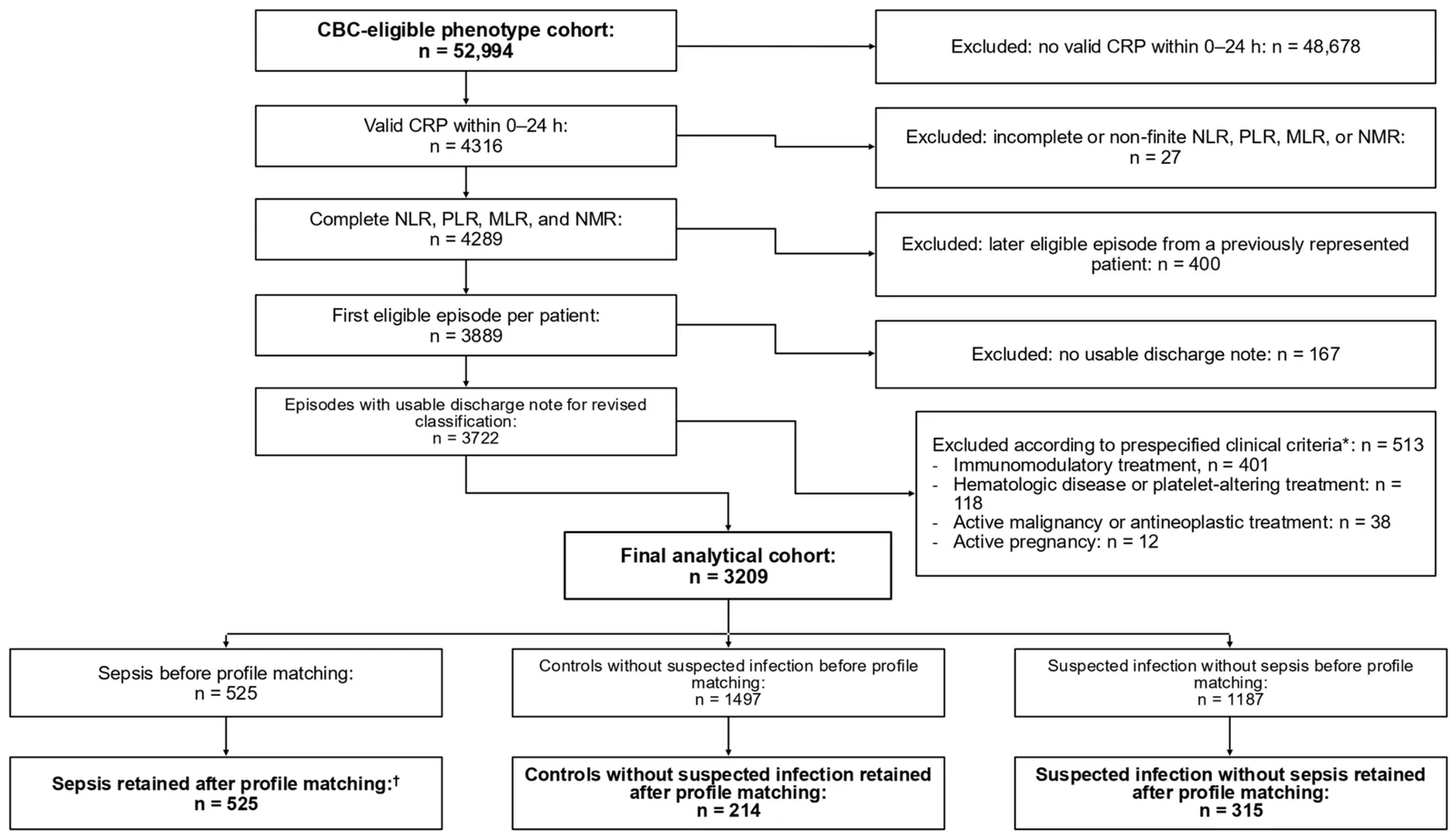
Participant flow from the CBC eligible cohort to the final analytical cohort and profile-matched comparison samples. * Prespecified clinical exclusion categories were not mutually exclusive; therefore, category-specific counts sum to more than the 513 episodes excluded on clinical criteria. †The same 525 sepsis episodes were retained as the target sepsis sample in both comparison-specific profile-matched analyses; the two comparator groups were matched separately. CBC, complete blood count; CRP, C-reactive protein; NLR, neutrophil-to-lymphocyte ratio; PLR, platelet-to-lymphocyte ratio; MLR, monocyte-to-lymphocyte ratio; NMR, neutrophil-to-monocyte ratio.

**Figure 2:**
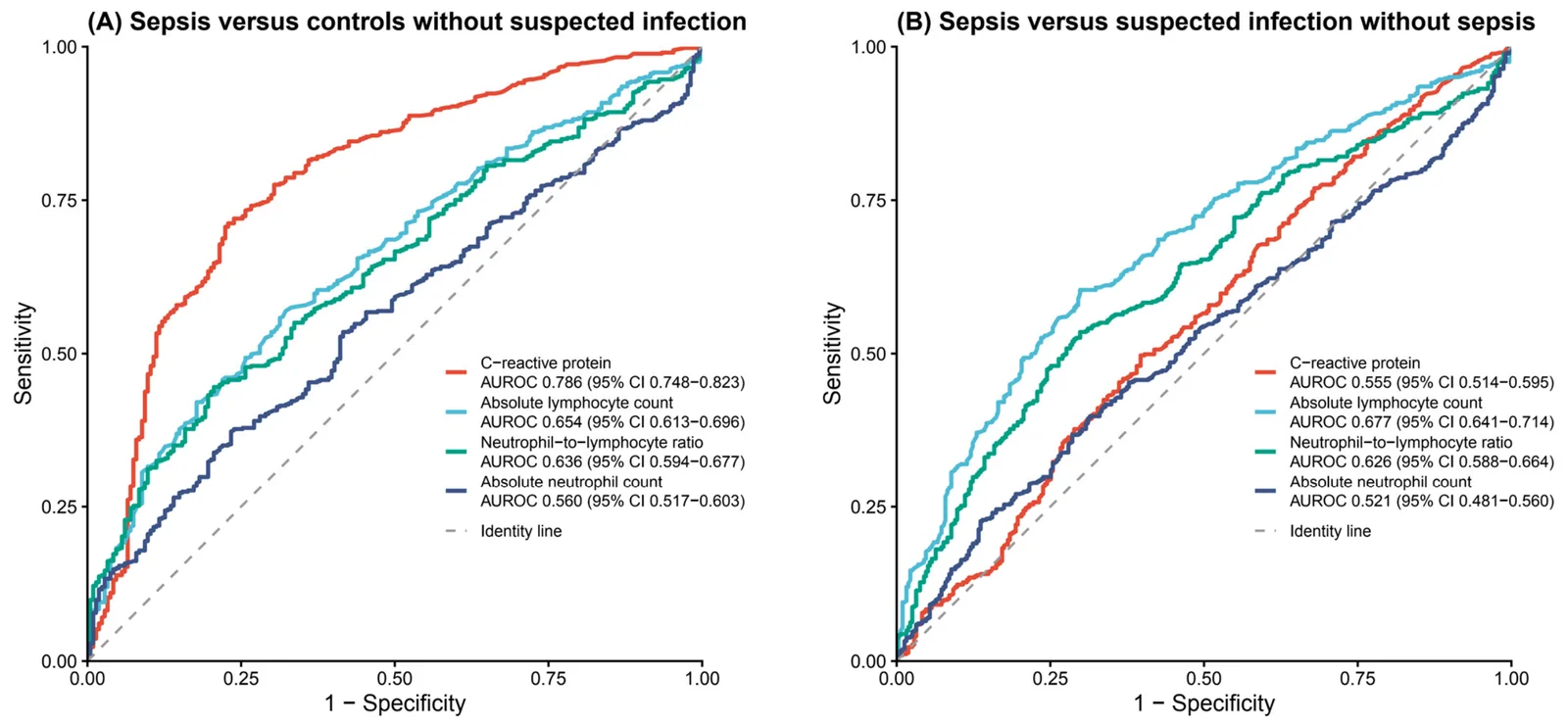
Receiver operating characteristic curves for selected biomarkers in the two matched comparison settings. Receiver operating characteristic curves are shown for C-reactive protein (CRP), absolute lymphocyte count, absolute neutrophil count and neutrophil-to-lymphocyte ratio (NLR). (**A**) Comparison of sepsis versus controls without suspected infection. (**B**) Comparison of sepsis versus suspected infection without sepsis. The same 525 sepsis episodes were included in both matched comparison settings. AUROC, area under the receiver operating characteristic curve; CI, confidence interval. The dashed diagonal line has been identified as the “Identity line”.

**Table 1:** Characteristics and covariate balance of the matched comparison samples.

Characteristic	Sepsis (n = 525)	Controls Without Suspected Infection (n = 214)	Absolute SMD	Suspected Infection Without Sepsis (n = 315)	Absolute SMD
Demographic characteristics					
Age, years	63.0 [52.0–73.0]	62.5 [40.5–82.0]	0.083	63.0 [43.0–78.5]	0.100
Female sex, n (%)	197 (37.5)	90 (42.1)	0.094	133 (42.2)	0.097
Race, n (%)					
White	337 (64.2)	128 (59.8)	0.091	217 (68.9)	0.098
Black	94 (17.9)	39 (18.2)	0.008	50 (15.9)	0.053
Hispanic/Latino	19 (3.6)	11 (5.1)	0.081	17 (5.4)	0.095
Asian	18 (3.4)	9 (4.2)	0.043	8 (2.5)	0.049
Other	28 (5.3)	11 (5.1)	0.009	12 (3.8)	0.068
Unknown	29 (5.5)	16 (7.5)	0.085	11 (3.5)	0.089
Insurance, n (%)					
Medicare	280 (53.3)	106 (49.5)	0.076	153 (48.6)	0.096
Medicaid	107 (20.4)	41 (19.2)	0.030	71 (22.5)	0.054
Private	125 (23.8)	58 (27.1)	0.077	77 (24.4)	0.015
Other	11 (2.1)	7 (3.3)	0.082	11 (3.5)	0.098
Unknown	2 (0.4)	2 (0.9)	0.090	3 (1.0)	0.093
Comorbidities					
Chronic pulmonary disease, n (%)	96 (18.3)	31 (14.5)	0.098	69 (21.9)	0.094
Liver disease, n (%)	106 (20.2)	35 (16.4)	0.096	51 (16.2)	0.100
Renal disease, n (%)	207 (39.4)	84 (39.3)	0.004	109 (34.6)	0.099
Diabetes, n (%)	202 (38.5)	72 (33.6)	0.099	106 (33.7)	0.099
Sepsis versus controls matching characteristics					
Observed noncoagulation SOFA category, n (%)				—	—
0	25 (4.8)	14 (6.5)	0.084	—	—
1	76 (14.5)	38 (17.8)	0.093	—	—
2–3	245 (46.7)	99 (46.3)	0.008	—	—
≥4	179 (34.1)	63 (29.4)	0.098	—	—
Unavailable noncoagulation SOFA components, median [IQR]	2 [1,2]	2 [1,2]	0.066	—	—
Sepsis versus suspected infection without sepsis matching characteristics					
Suspected infection site, n (%)		—	—		
Unknown	151 (28.8)	—	—	78 (24.8)	0.088
Skin/soft-tissue	89 (17.0)	—	—	62 (19.7)	0.073
Bone/joint	79 (15.0)	—	—	58 (18.4)	0.094
Gastrointestinal/intra-abdominal	25 (4.8)	—	—	21 (6.7)	0.089
Device/procedure related	17 (3.2)	—	—	15 (4.8)	0.086
Urinary	34 (6.5)	—	—	21 (6.7)	0.008
Respiratory	42 (8.0)	—	—	17 (5.4)	0.096
Bloodstream/endovascular	31 (5.9)	—	—	12 (3.8)	0.089
Multiple sites	34 (6.5)	—	—	13 (4.1)	0.096
Other less frequent	23 (4.4)	—	—	18 (5.7)	0.065

Values are median [IQR] or n (%), as indicated. SMD, standardized mean difference; SOFA, Sequential Organ Failure Assessment. SMD values are absolute post matching standardized mean differences. SMDs are displayed to three decimal places; values displayed as 0.100 reflect rounding, and no modeled balance term met or exceeded the prespecified absolute SMD threshold of 0.10 in the unrounded diagnostics. The same 525 sepsis episodes were included in both matched comparison settings. Comparison-specific matching characteristics are shown only for the setting in which they were used.

**Table 2:** Biomarker discrimination in the two matched comparison settings.

Biomarker	Sepsis Versus Controls Without Suspected Infection (n = 739)	Sepsis Versus Suspected Infection Without Sepsis (n = 840)
CRP	0.786 [0.748–0.823]	0.555 [0.514–0.595]
WBC	0.535 [0.492–0.578]	0.490 [0.451–0.529]
ANC	0.560 [0.517–0.603]	0.521 [0.481–0.560]
ALC	0.654 [0.613–0.696]	0.677 [0.641–0.714]
AMC	0.497 [0.453–0.541]	0.428 [0.389–0.467]
NLR	0.636 [0.594–0.677]	0.626 [0.588–0.664]
PLR	0.571 [0.528–0.614]	0.499 [0.460–0.538]
MLR	0.630 [0.587–0.672]	0.591 [0.552–0.630]
NMR	0.567 [0.523–0.611]	0.579 [0.540–0.618]
SIRI	0.605 [0.563–0.647]	0.559 [0.520–0.598]
SII	0.573 [0.530–0.616]	0.507 [0.468–0.547]

Values are AUROC [95% CI]. Confidence intervals were estimated using the DeLong method. The same 525 sepsis episodes were included in both matched comparison settings. Higher biomarker values were oriented toward sepsis for all biomarkers except absolute lymphocyte count, for which lower values were oriented toward sepsis. ANC, absolute neutrophil count; ALC, absolute lymphocyte count; AMC, absolute monocyte count; AUROC, area under the receiver operating characteristic curve; CI, confidence interval; CRP, C-reactive protein; NLR, neutrophil-to-lymphocyte ratio; PLR, platelet-to-lymphocyte ratio; MLR, monocyte-to-lymphocyte ratio; NMR, neutrophil-to-monocyte ratio; SIRI, Systemic Inflammation Response Index; SII, Systemic Immune Inflammation Index; WBC, white blood cell count. The dashed diagonal line has been identified as the “Identity line”.

**Table 3:** Differences in biomarker discrimination across clinical comparisons.

Biomarker	Sepsis Versus Controls Without Suspected Infection AUROC	Sepsis Versus Suspected Infection Without Sepsis AUROC	ΔAUROC [95% CI]	*p* Value	P_Holm-adj._ Value
CRP	0.786	0.555	**0.231 [0.183–0.278]**	<0.001	**<0.001**
WBC	0.535	0.490	0.045 [0.004–0.087]	0.033	0.228
ANC	0.560	0.521	0.039 [−0.004–0.082]	0.076	0.382
ALC	0.654	0.677	−0.023 [−0.065–0.019]	0.282	0.845
AMC	0.497	0.428	**0.069 [0.026–0.112]**	0.002	**0.014**
NLR	0.636	0.626	0.010 [−0.033–0.052]	0.657	1.000
PLR	0.571	0.499	**0.072 [0.029–0.114]**	0.001	**0.010**
MLR	0.630	0.591	0.038 [−0.006–0.083]	0.088	0.382
NMR	0.567	0.579	−0.012 [−0.057–0.034]	0.611	1.000
SIRI	0.605	0.559	0.046 [0.003–0.089]	0.037	0.228
SII	0.573	0.507	**0.066 [0.023–0.108]**	0.003	**0.021**

ΔAUROC was defined as the AUROC for sepsis versus controls without suspected infection minus the AUROC for sepsis versus suspected infection without sepsis. Positive values therefore indicate greater discrimination in the sepsis-versus-controls-without-suspected-infection setting. The same 525 sepsis episodes were included in both comparison settings, whereas the comparator samples were disjoint. *p* values are from two-sided Wald tests. Holm adjustment was applied across the 11 biomarker-specific cross-setting comparisons. ANC, absolute neutrophil count; ALC, absolute lymphocyte count; AMC, absolute monocyte count; AUROC, area under the receiver operating characteristic curve; CI, confidence interval; CRP, C-reactive protein; NLR, neutrophil-to-lymphocyte ratio; PLR, platelet-to-lymphocyte ratio; MLR, monocyte-to-lymphocyte ratio; NMR, neutrophil-to-monocyte ratio; SIRI, Systemic Inflammation Response Index; SII, Systemic Immune Inflammation Index; WBC, white blood-cell count. Bold values indicate statistically significant differences after Holm adjustment (adjusted *p* < 0.05).

## Data Availability

The data analyzed in this study are available through PhysioNet as part of the MIMIC-IV, MIMIC-IV-ED, and MIMIC-IV-Note databases, subject to the applicable credentialing, training, and Data Use Agreement requirements. No new patient-level dataset was generated for public distribution as part of this study. The analysis code supporting the reported results can be found at https://github.com/Sergio-ADC/sepsis_comparison_biomarker (accessed on 7 September 2026).

## Supplementary Materials

The following supporting information can be downloaded at https://www.mdpi.com/article/10.3390/jcm15218489/s1. Table S1: Clinical characteristics, organ dysfunction, and arrival physiology in the matched comparison samples; Table S2: Hematologic, inflammatory, and derived biomarker characteristics in the matched comparison samples; Table S3: AUROC estimates in the unmatched sensitivity analyses; Table S4: AUROC estimates in the coagulation sensitivity analyses; Table S5: Descriptive microbiologic characteristics of the matched suspected infection comparison sample; Table S6: Post hoc component analysis of the neutrophil-to-lymphocyte ratio using natural cubic splines in the matched comparison samples; Table S7: Clinical and laboratory characteristics by CRP eligibility in the post hoc sensitivity analysis; Table S8: Unmatched AUROC estimates for CBC biomarkers by CRP eligibility in the post hoc sensitivity analysis; Figure S1: Covariate balance before and after profile matching in the primary and coagulation sensitivity analyses; Figure S2: Pairwise differences in biomarker discrimination in the primary matched analyses; Figure S3: Pairwise differences in biomarker discrimination in the unmatched sensitivity analyses; Figure S4: Pairwise differences in biomarker discrimination in the coagulation sensitivity analyses; and Figure S5: Nonlinear associations of absolute lymphocyte and neutrophil counts with sepsis in the matched comparison samples.

## Abbreviations

The following abbreviations are used in this manuscript:

ALC	Absolute lymphocyte count
AMC	Absolute monocyte count
ANC	Absolute neutrophil count
ATT	Average treatment effect on the treated
AUROC	Area under the receiver operating characteristic curve
CBC	Complete blood count
CI	Confidence interval
CRP	C-reactive protein
ED	Emergency department
IQR	Interquartile range
MIMIC-IV	Medical Information Mart for Intensive Care IV
MIMIC-IV-ED	MIMIC-IV Emergency Department
MIMIC-IV-Note	MIMIC-IV clinical notes database
MLR	Monocyte-to-lymphocyte ratio
NLR	Neutrophil-to-lymphocyte ratio
NMR	Neutrophil-to-monocyte ratio
PLR	Platelet-to-lymphocyte ratio
ROC	Receiver operating characteristic
SII	Systemic Immune-Inflammation Index
SIRI	Systemic Inflammation Response Index
SMD	Standardized mean difference
SOFA	Sequential Organ Failure Assessment
SvC	Sepsis versus controls without suspected infection
SvI	Sepsis versus suspected infection without sepsis
WBC	White blood cell count
