# Supplementary material for: Clinical Comparator Dependence of Inflammatory and Hematologic Biomarker Discrimination for Sepsis: A Matched Retrospective Study Using MIMIC-IV Emergency Department Data
Source: J Clin Med. 2026 Nov 1;15(21):8489. doi: 10.3390/jcm15218489 (PMC13764433; doi:10.3390/jcm15218489)
Supplement: Supplementary file 1 [file jcm-15-08489-s001.zip › jcm-4581750-supplementary.pdf]

Supplementary material.

Supplementary methods.

Post hoc analysis of NLR components.

Because of the relative performance of the neutrophil to lymphocyte ratio (NLR) and absolute lymphocyte count in the primary discrimination analyses, we conducted a post hoc analysis to examine the relative statistical contributions of the two cell count components of NLR. Within each primary matched comparison sample, logistic regression models were fitted using absolute lymphocyte count alone, absolute neutrophil count alone, or both components. Absolute lymphocyte and neutrophil counts were retained as continuous predictors and modeled using natural cubic splines, allowing their associations with the log-odds of sepsis to vary smoothly across the observed range rather than imposing a constant linear association or categorizing the predictors. Cubic spline approaches have been described for flexible modeling of continuous predictors in logistic and other regression models and for avoiding restrictive linearity or categorization assumptions [34].

Three spline degrees of freedom per component were used for the main specification, and four degrees of freedom were examined as a sensitivity analysis. Likelihood-ratio tests compared the combined lymphocyte plus neutrophil model with each corresponding single-component model. These comparisons assessed the additional statistical contribution of lymphocyte count after accounting for neutrophil count and, conversely, the additional contribution of neutrophil count after accounting for lymphocyte count.

To account for multiple testing, Holm procedure was applied across the four directional component comparisons within each spline specification. Thus, the four tests from the 3 degree of freedom specification constituted one multiplicity family, and the four tests from the 4 degree of freedom sensitivity specification constituted a separate family [32]. Changes in Akaike information criterion (AIC), Bayesian information criterion (BIC), and apparent area under the receiver operating characteristic curve (AUROC) were examined descriptively. For AIC and BIC, differences were calculated as the value for the combined lymphocyte plus neutrophil model minus that of the corresponding single-component model, such that negative values favored the combined model. Apparent AUROCs were calculated in the same matched samples used for model fitting and were therefore treated as descriptive measures of in sample discrimination rather than validated estimates of incremental predictive performance.

For visualization, fitted associations from the combined models were displayed over the central 95% of observed values for each cell count component. Each association was centered at the median of the focal predictor while the other component was held at its sample median. The primary 3 degree of freedom and 4 degree of freedom sensitivity specifications were displayed together, with 95% Wald confidence intervals calculated for the

corresponding centered contrasts (Supplementary Figure S5). Detailed model comparisons are reported in Supplementary Table S6.

Supplementary Table S1. Clinical characteristics, organ dysfunction, and arrival physiology in the matched comparison samples.

Characteristic	Sepsis (n=525)	Controls without suspected infection (n=214)	Absolute SMD	Suspected infection without sepsis (n=315)	Absolute SMD
**Organ dysfunction**					
Total observed SOFA score	3.0 [2.0–5.0] (525/525)	3.0 [2.0–4.0] (214/214)	0.412	1.0 [0.0–1.0] (315/315)	1.420
Observed noncoagulation SOFA score	3.0 [2.0–4.0] (525/525)	2.0 [2.0–4.0] (214/214)	0.258	0.0 [0.0–1.0] (315/315)	1.230
Respiratory SOFA score	2.0 [2.0–3.0] (30/525)	2.0 [1.0–2.0] (7/214)	NA	NA (0/315)	NA
Coagulation SOFA score	0.0 [0.0–1.0] (525/525)	0.0 [0.0–0.0] (214/214)	0.418	0.0 [0.0–0.0] (315/315)	0.666
Hepatic SOFA score	0.0 [0.0–1.0] (413/525)	0.0 [0.0–1.0] (178/214)	NA	0.0 [0.0–0.0] (181/315)	NA
Cardiovascular SOFA score	1.0 [0.0–1.0] (525/525)	0.0 [0.0–1.0] (213/214)	0.418	0.0 [0.0–0.0] (314/315)	0.660
Neurologic SOFA score	1.0 [0.0–1.0] (208/525)	1.0 [0.0–2.0] (71/214)	NA	0.0 [0.0–0.0] (22/315)	NA
Renal SOFA score	1.0 [0.0–2.0] (525/525)	1.0 [0.0–2.0] (213/214)	0.075	0.0 [0.0–1.0] (315/315)	0.833
**Arrival physiology**					
Temperature at ED arrival, °C	36.9 [36.6–37.4] (505/525)	36.7 [36.4–36.9] (200/214)	0.427	36.8 [36.6–37.2] (308/315)	0.076
Heart rate at ED arrival, beats/min	88 [76–100] (510/525)	82 [72–96] (204/214)	0.263	90 [74–102] (310/315)	0.013
Respiratory rate at ED arrival, breaths/min	18 [16–20] (510/525)	18 [16–18] (203/214)	0.291	18 [16–18] (309/315)	0.195
Mean arterial pressure at ED arrival, mmHg	87 [76–101] (510/525)	94 [83–107] (203/214)	0.363	95 [85–106] (310/315)	0.350
SpO_2 at ED arrival, %	98 [96–100] (503/525)	99 [97–100] (203/214)	0.230	98 [97–100] (308/315)	0.106
**Organ function laboratory tests**					
First creatinine within 0–24 h, mg/dL	1.40 [1.00–2.50] (525/525)	1.30 [0.90–2.50] (213/214)	0.012	1.00 [0.80–1.20] (315/315)	0.529
First total bilirubin within 0–24 h, mg/dL	0.60 [0.40–1.20] (413/525)	0.60 [0.30–1.10] (178/214)	NA	0.40 [0.30–0.60] (181/315)	NA

Values are median [IQR] followed by the number of episodes with available data/total number of episodes, unless otherwise indicated. SMDs are absolute standardized mean differences and are presented descriptively for the variables shown; they should not be interpreted as matching-balance diagnostics. Profile matching used prespecified modeled terms, including categories of observed noncoagulation SOFA and the number of unavailable noncoagulation SOFA components rather than the continuous SOFA scores displayed here. The same 525 sepsis episodes were included in both matched comparison settings. SOFA component scores reflect observed revised component information available within 0–24 h; unavailable components were not assigned additional points to the observed score. NA indicates that the corresponding value or standardized mean difference was not available or not calculated. ED, emergency department; IQR, interquartile range; SMD, standardized mean difference; SOFA, Sequential Organ Failure Assessment; SpO_2, peripheral oxygen saturation.

Supplementary Table S2. Hematologic, inflammatory, and derived biomarker characteristics in the matched comparison samples.

Characteristic	Sepsis (n=525)	Controls without suspected infection (n=214)	Absolute SMD	Suspected infection without sepsis (n=315)	Absolute SMD
**Hematology**					
Hemoglobin, g/dL	10.5 [8.9–12.1] (524/525)	11.6 [10.2–13.1] (214/214)	0.459	11.2 [9.9–12.8] (315/315)	0.314
Platelet count, $\times10^9$/L	175 [124–253] (525/525)	210 [161–276] (214/214)	0.271	256 [201–324] (315/315)	0.660
WBC, $\times10^9$/L	9.30 [5.90–13.20] (525/525)	8.60 [6.43–11.17] (214/214)	0.196	9.60 [6.90–12.50] (315/315)	0.051
ANC, $\times10^9$/L	6.96 [4.24–10.92] (525/525)	5.97 [3.89–8.71] (214/214)	0.254	6.61 [4.33–9.65] (315/315)	0.113
ALC, $\times10^9$/L	1.01 [0.64–1.52] (525/525)	1.37 [0.95–2.03] (214/214)	0.041	1.46 [1.06–2.09] (315/315)	0.065
AMC, $\times10^9$/L	0.59 [0.38–0.93] (525/525)	0.61 [0.42–0.81] (214/214)	0.066	0.69 [0.51–0.92] (315/315)	0.129
**Inflammatory and derived biomarkers**					
CRP, mg/L	76.4 [25.3–143.2] (525/525)	9.6 [3.1–32.6] (214/214)	0.793	61.6 [14.2–124.2] (315/315)	0.157
NLR	6.48 [3.58–12.36] (525/525)	4.38 [2.57–7.15] (214/214)	0.346	4.46 [2.61–6.97] (315/315)	0.314
PLR	174.77 [109.68–298.82] (525/525)	145.86 [94.67–216.76] (214/214)	0.230	173.81 [116.51–256.71] (315/315)	0.114
MLR	0.58 [0.36–1.00] (525/525)	0.42 [0.29–0.65] (214/214)	0.370	0.46 [0.30–0.71] (315/315)	0.274
NMR	10.66 [7.30–16.98] (525/525)	9.41 [6.55–12.85] (214/214)	0.117	9.03 [6.51–12.79] (315/315)	0.153
SIRI	3.78 [1.73–9.13] (525/525)	2.54 [1.28–4.67] (214/214)	0.313	2.93 [1.36–6.01] (315/315)	0.209
SII	1177.6 [528.3–2655.5] (525/525)	856.1 [483.0–1634.0] (214/214)	0.237	1081.5 [613.4–2075.3] (315/315)	0.122

Values are median [IQR] followed by the number of episodes with available data/total number of episodes. SMDs are absolute standardized mean differences and are presented

descriptively; the biomarkers shown in this table were not matching targets and these SMDs should not be interpreted as matching-balance diagnostics. The same 525 sepsis episodes were included in both matched comparison settings. Biomarker measurements were derived from the prespecified 0–24 h window after emergency department arrival. The index complete blood count specimen provided platelet, neutrophil, lymphocyte, and monocyte counts from the same specimen; CRP was selected within the same 0–24 h window according to the prespecified temporal selection rule. ANC, absolute neutrophil count; ALC, absolute lymphocyte count; AMC, absolute monocyte count; IQR, interquartile range; SMD, standardized mean difference; CRP, C-reactive protein; NLR, neutrophil to lymphocyte ratio; PLR, platelet to lymphocyte ratio; MLR, monocyte to lymphocyte ratio; NMR, neutrophil to monocyte ratio; SIRI, Systemic Inflammation Response Index; SII, Systemic Immune Inflammation Index; WBC, white blood-cell count.

Supplementary Table S3. AUROC estimates in the unmatched sensitivity analyses.

Biomarker	Sepsis vs controls without suspected infection (n=2,022)	Sepsis vs suspected infection without sepsis (n=1,712)
CRP	0.810 [0.790–0.831]	0.570 [0.542–0.599]
WBC	0.558 [0.526–0.590]	0.483 [0.451–0.515]
ANC	0.608 [0.577–0.638]	0.515 [0.484–0.547]
ALC	0.742 [0.717–0.768]	0.692 [0.664–0.720]
AMC	0.509 [0.478–0.540]	0.457 [0.426–0.488]
NLR	0.725 [0.698–0.752]	0.629 [0.600–0.659]
PLR	0.597 [0.566–0.628]	0.494 [0.463–0.526]
MLR	0.715 [0.688–0.741]	0.625 [0.596–0.655]
NMR	0.596 [0.567–0.626]	0.545 [0.514–0.575]
SIRI	0.684 [0.655–0.712]	0.577 [0.547–0.608]
SII	0.617 [0.587–0.648]	0.498 [0.467–0.530]

Values are AUROC [95% CI]. The unmatched sepsis versus controls without suspected infection analysis included 525 sepsis episodes and 1,497 comparator episodes (n=2,022). The unmatched sepsis versus suspected infection without sepsis analysis included the same 525 sepsis episodes and 1,187 comparator episodes (n=1,712). Confidence intervals were calculated using the DeLong method. ROC direction was prespecified according to the clinical direction of each biomarker: lower absolute lymphocyte counts indicated sepsis, whereas higher values indicated sepsis for all other biomarkers. This sensitivity analysis is descriptive; no formal statistical tests were performed to compare matched and unmatched AUROC estimates. ANC, absolute neutrophil count; ALC, absolute lymphocyte count; AMC, absolute monocyte count; AUROC, area under the receiver operating characteristic curve; CI, confidence interval; CRP, C-reactive protein; NLR, neutrophil to lymphocyte ratio; PLR, platelet to lymphocyte ratio; MLR, monocyte to lymphocyte ratio; NMR, neutrophil to monocyte ratio; SIRI, Systemic Inflammation Response Index; SII, Systemic Immune Inflammation Index; WBC, white blood-cell count.

Supplementary Table S4. AUROC estimates in the coagulation sensitivity analyses.

Biomarker	Sepsis vs controls without suspected infection (n=590)	Sepsis vs suspected infection without sepsis (n=694)
CRP	0.753 [0.707–0.800]	0.561 [0.517–0.605]
WBC	0.559 [0.510–0.607]	0.512 [0.469–0.555]
ANC	0.577 [0.529–0.626]	0.541 [0.498–0.584]
ALC	0.619 [0.571–0.667]	0.658 [0.618–0.699]
AMC	0.498 [0.449–0.548]	0.451 [0.408–0.493]
NLR	0.635 [0.589–0.681]	0.630 [0.589–0.672]
PLR	0.597 [0.548–0.645]	0.520 [0.477–0.563]
MLR	0.609 [0.561–0.657]	0.592 [0.550–0.635]
NMR	0.574 [0.524–0.624]	0.574 [0.532–0.617]
SIRI	0.606 [0.559–0.654]	0.573 [0.530–0.616]
SII	0.606 [0.558–0.653]	0.533 [0.490–0.576]

Values are AUROC [95% CI]. The coagulation sensitivity analysis excluded sepsis episodes whose sepsis classification depended on points contributed by the coagulation component of SOFA, leaving 424 sepsis episodes. Comparison groups were then rematched separately within each comparison setting. The sepsis versus controls without suspected infection analysis included 424 sepsis episodes and 166 comparator episodes (n=590), whereas the sepsis versus suspected infection without sepsis analysis included 424 sepsis episodes and 270 comparator episodes (n=694). For the sepsis versus controls without suspected infection comparison, the comparator candidate pool was additionally restricted to noncoagulation SOFA categories represented among the retained sepsis episodes before rematching (this restriction did not remove additional sepsis episodes). The sepsis versus suspected infection without sepsis comparison, the comparator candidate pool was otherwise retained according to the original specification. Confidence intervals were calculated using the DeLong method. ROC direction was prespecified according to the clinical direction of each biomarker: lower absolute lymphocyte counts indicated sepsis, whereas higher values indicated sepsis for all other biomarkers. Matching was repeated separately for this sensitivity analysis, and AUROC estimates are presented descriptively. ANC, absolute neutrophil count; ALC, absolute lymphocyte count; AMC, absolute monocyte count; AUROC, area under the receiver operating characteristic curve; CI, confidence interval; CRP, C-reactive protein; NLR, neutrophil to lymphocyte ratio; PLR, platelet to lymphocyte ratio; MLR, monocyte to lymphocyte ratio; NMR, neutrophil to monocyte ratio; SIRI, Systemic Inflammation Response Index; SII, Systemic Immune Inflammation Index; WBC, white blood cell count.

Supplementary Table S5. Descriptive microbiologic characteristics of the matched suspected infection comparison sample.

Characteristic	Sepsis (n=525)	Suspected infection without sepsis (n=315)	Absolute SMD
**Microbiologic sampling and yield**			
Qualifying microbiology specimens per episode, median [IQR]	3 [3–5]	3 [2–5]	0.220
Distinct microbiology studies per episode, median [IQR]	3 [2–5]	2 [1–4]	0.122
At least one valid reported microbiologic finding, n (%)	211 (40.2%)	117 (37.1%)	0.062
Distinct microbiologic findings per microbiology positive episode, median (IQR)	1 [1–2]	1 [1–2]	—
Multiple distinct microbiologic findings, n (% of microbiology positive episodes)	75 (35.5%)	42 (35.9%)	—
**Blood cultures**			
At least one blood culture obtained, n (%)	482 (91.8%)	289 (91.7%)	0.002
At least one valid positive blood culture, n (%)	92 (17.5%)	34 (10.8%)	0.177
Blood-culture positivity among cultured episodes, n/N (%)	92/482 (19.1%)	34/289 (11.8%)	—
**Most frequent reported microbiologic findings**			
Staphylococcus aureus	56 (10.7%)	39 (12.4%)	0.056
Mixed bacterial microbiota	27 (5.1%)	24 (7.6%)	0.112
Coagulase-negative staphylococci	37 (7.0%)	9 (2.9%)	0.164
Escherichia coli	28 (5.3%)	16 (5.1%)	0.011
Enterococcus spp.	20 (3.8%)	11 (3.5%)	0.017
Yeast	15 (2.9%)	11 (3.5%)	0.038
Group B *Streptococcus*	15 (2.9%)	10 (3.2%)	0.019
Corynebacterium spp.	16 (3.0%)	5 (1.6%)	0.085
Pseudomonas aeruginosa	11 (2.1%)	7 (2.2%)	0.009
Clostridioides difficile	9 (1.7%)	5 (1.6%)	0.010
Proteus mirabilis	8 (1.5%)	6 (1.9%)	0.031

Values are median [IQR], n (%), or n/N (%), as indicated. The matched comparison included 525 sepsis episodes and 315 episodes with suspected infection without sepsis. SMDs for episode-level continuous and binary characteristics were calculated across all matched episodes. SMDs are not reported for characteristics conditioned on microbiology positive or cultured episodes. A microbiology positive episode was defined as an episode with at least one valid reported microbiologic finding after exclusion of a cancelled result. For the most frequent microbiologic findings, percentages and SMDs use all matched episodes in the corresponding group as denominators; findings are not mutually exclusive. Coagulase-negative staphylococci include generic coagulase-negative staphylococcal findings and *Staphylococcus epidermidis*, whereas *Staphylococcus lugdunensis* was retained separately.

All Enterococcus identifications were grouped as *Enterococcus* spp. IQR, interquartile range; SMD, standardized mean difference.

Supplementary Table S6. Post hoc component analysis of the neutrophil to lymphocyte ratio using natural cubic splines in the matched comparison samples.

Spline df	Added component	Comparison setting	LRT χ^2	Holm adjusted P	ΔAIC	ΔBIC	Apparent AUROC, reduced → combined (Δ)
**3**	**ALC after accounting for ANC**	Sepsis vs controls without suspected infection	56.4	**<0.001**	−50.4	−36.6	0.591 → 0.700 (0.109)
		Sepsis vs suspected infection without sepsis	90.9	**<0.001**	−84.9	−70.7	0.577 → 0.707 (0.130)
	**ANC after accounting for ALC**	Sepsis vs controls without suspected infection	19.3	**<0.001**	−13.3	+0.514	0.673 → 0.700 (0.027)
		Sepsis vs suspected infection without sepsis	8.21	**0.042**	−2.21	+12.0	0.696 → 0.707 (0.011)
**4**	**ALC after accounting for ANC**	Sepsis vs controls without suspected infection	55.6	**<0.001**	−47.6	−29.1	0.603 → 0.705 (0.102)
		Sepsis vs suspected infection without sepsis	91.0	**<0.001**	−83.0	−64.1	0.580 → 0.707 (0.128)
	**ANC after accounting for ALC**	Sepsis vs controls without suspected infection	20.8	**<0.001**	−12.8	+5.65	0.673 → 0.705 (0.032)
		Sepsis vs suspected infection without sepsis	9.10	0.059	−1.10	+17.8	0.696 → 0.707 (0.012)

This post hoc analysis used the primary matched samples: 525 sepsis episodes and 214 controls without suspected infection (n=739), and the same 525 sepsis episodes and 315 episodes with suspected infection without sepsis (n=840). Absolute lymphocyte count (ALC) and absolute neutrophil count (ANC) were modeled as continuous variables using natural cubic splines. Three degrees of freedom per component were used for the primary specification, and four degrees of freedom were examined as a sensitivity specification. Likelihood-ratio tests compared the combined lymphocyte plus neutrophil model with the corresponding single-component model. Holm adjustment was applied across the four

directional component comparisons within each spline specification. ΔAIC and ΔBIC are calculated as the information criterion for the combined model minus that for the corresponding reduced single-component model (negative values favors the combined model, positive values favor the reduced model). Apparent AUROCs were calculated in the same samples used for model fitting and are presented only as descriptive measures of in sample discrimination; they should not be interpreted as validated estimates of incremental predictive performance. AIC, Akaike information criterion; AUROC, area under the receiver operating characteristic curve; BIC, Bayesian information criterion; df, degrees of freedom; LRT, likelihood-ratio test.

Supplementary Table S7. Clinical and laboratory characteristics by CRP eligibility in the post hoc sensitivity analysis.

Characteristic	CRP-eligible (n=3,889)	CBC-only (n=34,200)	Absolute SMD
**Clinical characteristics**			
Age, years	59 [44–71]	62 [47–76]	0.161
Female sex, n (%)	1,973 (50.7%)	17,744 (51.9%)	0.023
Charlson comorbidity index	3 [1–5]	3 [1–6]	0.134
ED admission, n (%)	3,698 (95.1%)	29,422 (86.0%)	0.314
Time from ED arrival to index CBC, h	3.15 [1.18–13.52]	2.10 [0.77–8.95]	0.230
**Phenotype classification**			
Controls without suspected infection, n (%)	1,873 (48.2%)	20,284 (59.3%)	—
Suspected infection without sepsis, n (%)	1,379 (35.5%)	7,134 (20.9%)	—
Sepsis, n (%)	637 (16.4%)	6,782 (19.8%)	—
**CBC measurements and derived index**			
WBC, $\times 10^9$/L	8.80 [6.50–11.60]	8.80 [6.40–12.00]	0.067
ANC, $\times 10^9$/L	5.98 [4.01–8.77]	6.11 [4.02–9.34]	0.080
ALC, $\times 10^9$/L	1.53 [0.99–2.17]	1.43 [0.90–2.10]	0.005
Platelet count, $\times 10^9$/L	238 [183–308]	222 [168–282]	0.212
NLR	3.90 [2.22–7.25]	4.20 [2.26–8.40]	0.083
**Structured SOFA assessment**			
Structured SOFA total	1 [0–2]	1 [0–2]	0.212
Number of available structured SOFA components	4 [3–4]	4 [3–4]	0.018

Values are median [IQR] or n (%). Proportions may not sum to 100% because of rounding. Each patient contributed one episode and selection prioritized the first episode with valid CRP within 0–24 h and complete, finite values for the four required CBC ratios. Among patients with no such episode, the first CBC episode with complete, finite ratios was selected. The 3,889 CRP-eligible episodes correspond to Figure 1 before clinical review and exclusions. Phenotypes were assigned using the automated suspected infection algorithm and structured SOFA scores. Unavailable SOFA components contributed no additional points. Clinical review, manual SOFA revision, and the clinical exclusions used in the primary analysis were not applied to either group. ALC, absolute lymphocyte count; ANC, absolute neutrophil count; CBC, complete blood count; CRP, C-reactive protein; ED, emergency department; IQR, interquartile range; NLR, neutrophil to lymphocyte ratio; SMD, standardized mean difference; SOFA, Sequential Organ Failure Assessment; WBC, white blood cell count.

Supplementary Table S8. Unmatched AUROC estimates for CBC biomarkers by CRP eligibility in the post hoc sensitivity analysis.

Biomarker	Sepsis vs controls without suspected infection		Sepsis vs suspected infection without sepsis	
	CRP-eligible (n=2,510)	CBC-only (n=27,066)	CRP-eligible (n=2,016)	CBC-only (n=13,916)
WBC	0.552 [0.523–0.581]	0.586 [0.577–0.595]	0.477 [0.448–0.506]	0.493 [0.483–0.503]
ANC	0.599 [0.571–0.627]	0.621 [0.612–0.629]	0.508 [0.479–0.537]	0.510 [0.500–0.519]
ALC	0.737 [0.713–0.760]	0.725 [0.718–0.732]	0.687 [0.662–0.713]	0.653 [0.644–0.662]
AMC	0.509 [0.480–0.537]	0.517 [0.508–0.525]	0.459 [0.430–0.488]	0.454 [0.445–0.464]
NLR	0.722 [0.698–0.746]	0.717 [0.710–0.725]	0.628 [0.601–0.655]	0.596 [0.586–0.605]
PLR	0.588 [0.560–0.616]	0.593 [0.584–0.601]	0.489 [0.461–0.518]	0.465 [0.455–0.475]
MLR	0.709 [0.684–0.733]	0.690 [0.682–0.697]	0.622 [0.594–0.649]	0.581 [0.572–0.591]
NMR	0.589 [0.562–0.616]	0.599 [0.591–0.608]	0.541 [0.512–0.569]	0.543 [0.534–0.553]
SIRI	0.679 [0.653–0.705]	0.675 [0.666–0.683]	0.573 [0.545–0.601]	0.550 [0.540–0.560]
SII	0.608 [0.580–0.635]	0.620 [0.611–0.629]	0.491 [0.462–0.519]	0.478 [0.468–0.487]

Values are AUROC [95% CI]; confidence intervals were estimated using the DeLong method.. The CRP-eligible analyses included 637 sepsis episodes and either 1,873 controls without suspected infection or 1,379 episodes with suspected infection without sepsis. Corresponding CBC-only analyses included 6,782 sepsis episodes and either 20,284 controls or 7,134 episodes with suspected infection without sepsis. Phenotypes were assigned automatically using suspected infection status and structured SOFA scores, with unavailable components contributing no additional points. Clinical review, manual SOFA revision, clinical exclusions, infection site adjudication and matching were not applied. ALC, absolute lymphocyte count; AMC, absolute monocyte count; ANC, absolute neutrophil count; AUROC, area under the receiver operating characteristic curve; CBC, complete blood count; CI, confidence interval; CRP, C-reactive protein; MLR, monocyte to lymphocyte ratio; NLR, neutrophil to lymphocyte ratio; NMR, neutrophil to monocyte ratio; PLR, platelet to lymphocyte ratio; ROC, receiver operating characteristic; SII, systemic immune inflammation index; SIRI, systemic inflammation response index; SOFA, Sequential Organ Failure Assessment; WBC, white blood cell count.

Supplementary Figure S1. Covariate balance before and after profile matching in the primary and coagulation sensitivity analyses.

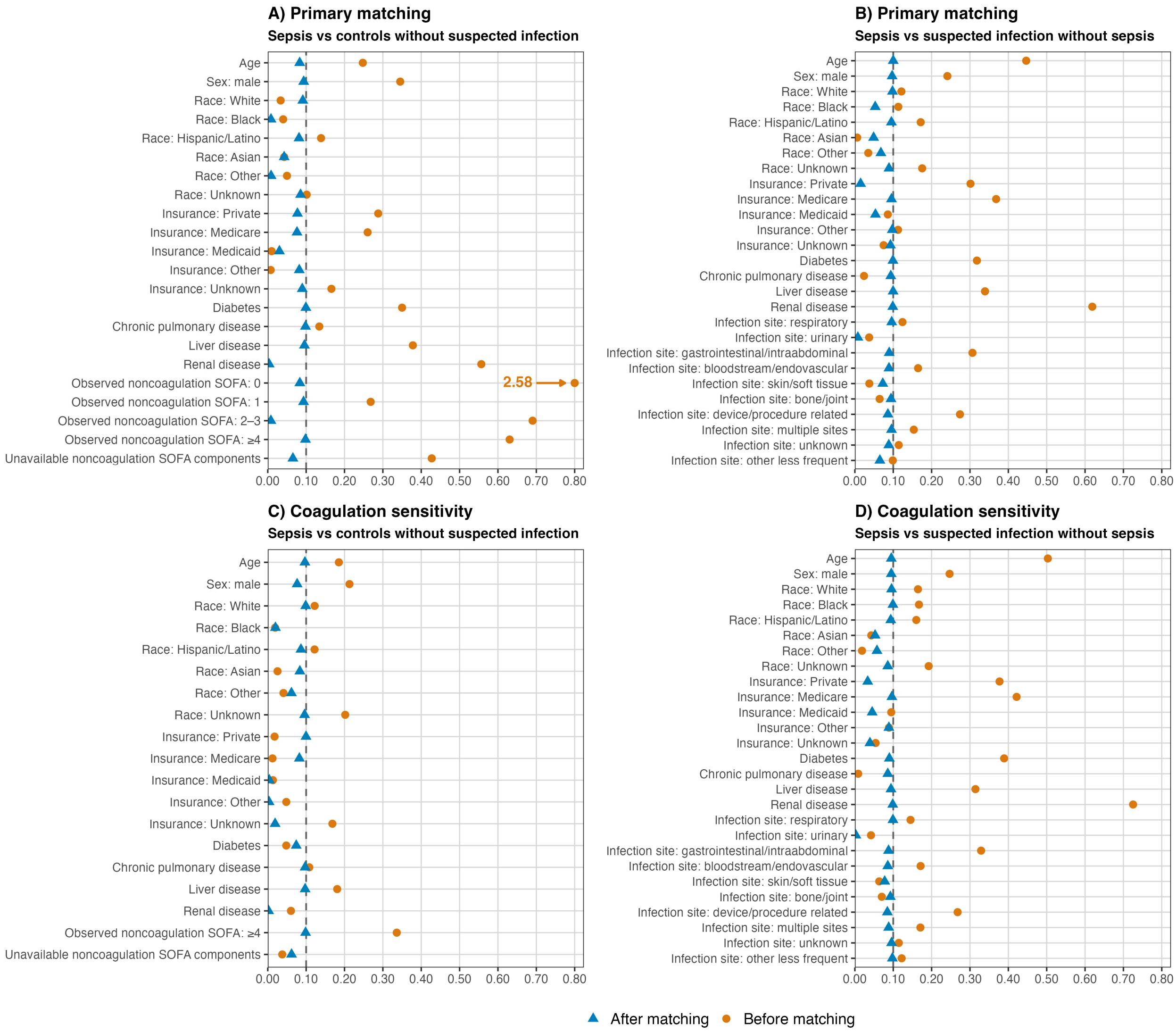

Absolute standardized mean differences are shown before matching (orange circles) and after matching (blue triangles). Panels A and B show the primary matched comparisons of sepsis versus controls without suspected infection and sepsis versus suspected infection without sepsis, respectively. Panels C and D show the corresponding rematched coagulation sensitivity analyses. The dashed vertical line indicates the prespecified absolute standardized mean difference threshold of 0.10. In Panel A, the largest pre-matching standardized difference exceeded the plotting range and is indicated by its numerical value. SMD, standardized mean difference.

Supplementary Figure S2. Pairwise differences in biomarker discrimination in the primary matched analyses.

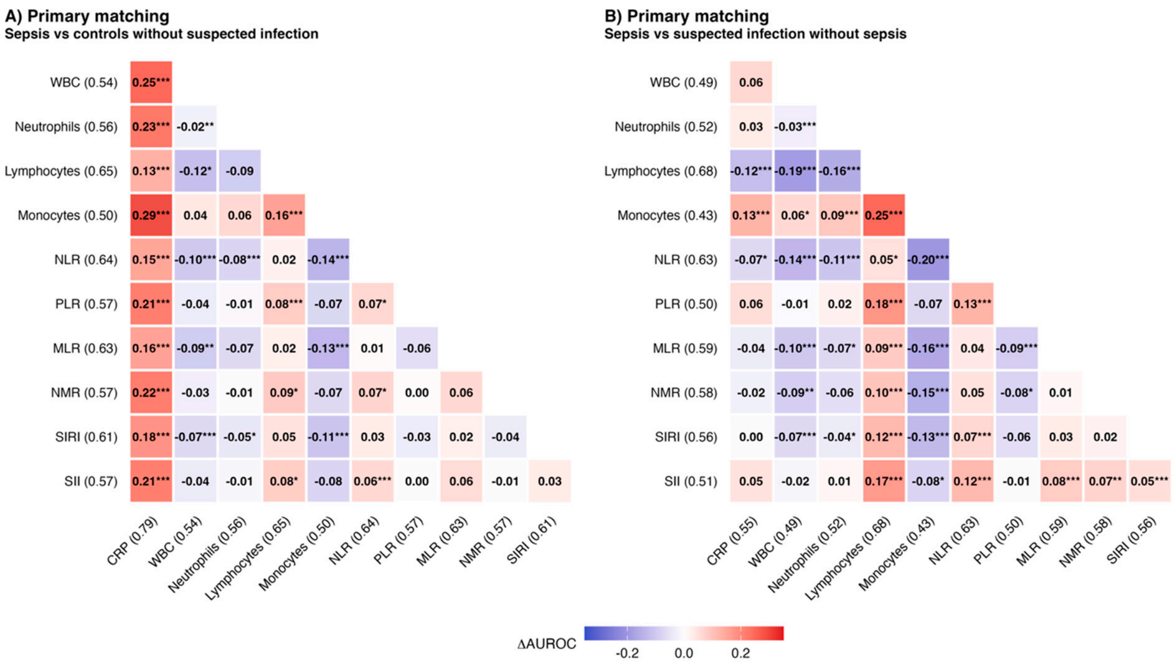

Panels A and B show the sepsis versus controls without suspected infection and sepsis versus suspected infection without sepsis comparisons, respectively. AUROC values for each biomarker are shown in parentheses in the axis labels. Each cell represents ΔAUROC = AUROC of the column biomarker minus AUROC of the row biomarker; positive values therefore indicate greater discrimination for the column biomarker and negative values greater discrimination for the row biomarker. Statistical significance was assessed using paired DeLong tests, with Holm adjustment across the 55 pairwise biomarker comparisons within each panel. *Holm adjusted P<0.05; **P<0.01; ***P<0.001. AUROC, area under the receiver operating characteristic curve.

Supplementary Figure S3. Pairwise differences in biomarker discrimination in the unmatched sensitivity analyses.

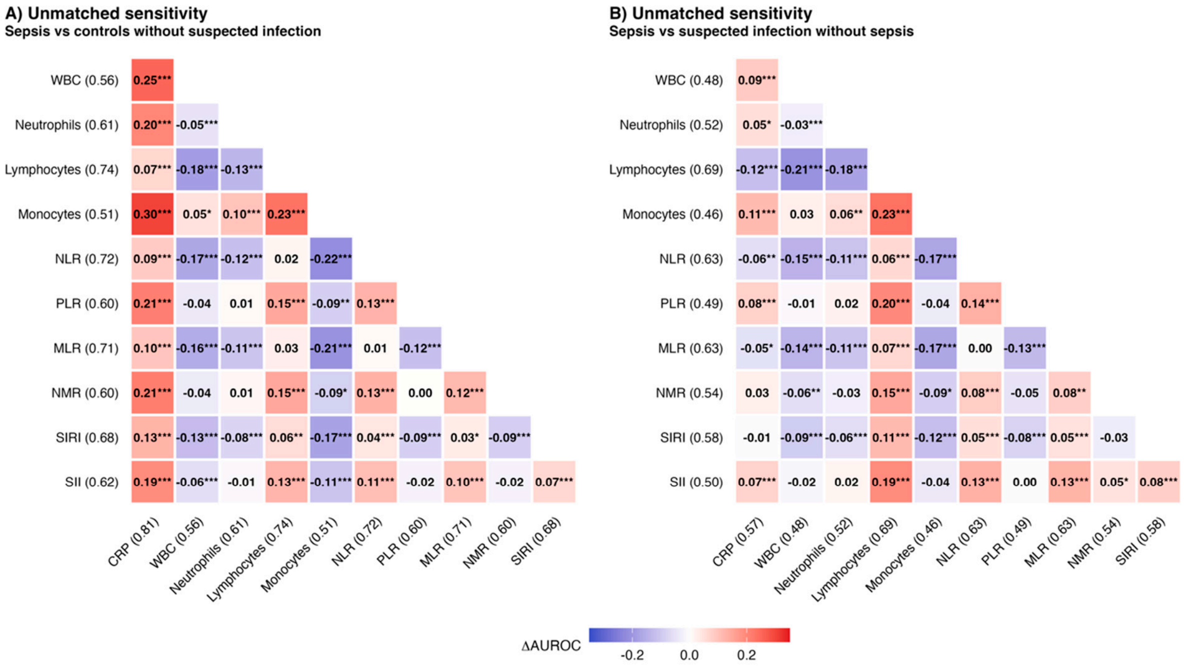

Panels A and B show the unmatched sepsis versus controls without suspected infection and sepsis versus suspected infection without sepsis comparisons, respectively. AUROC values for each biomarker are shown in parentheses in the axis labels. Each cell represents ΔAUROC = AUROC of the column biomarker minus AUROC of the row biomarker; positive values indicate greater discrimination for the column biomarker and negative values greater discrimination for the row biomarker. Statistical significance was assessed using paired DeLong tests, with Holm adjustment across the 55 pairwise biomarker comparisons within each panel. *Holm adjusted P<0.05; **P<0.01; ***P<0.001. AUROC, area under the receiver operating characteristic curve.

Supplementary Figure S4. Pairwise differences in biomarker discrimination in the coagulation sensitivity analyses.

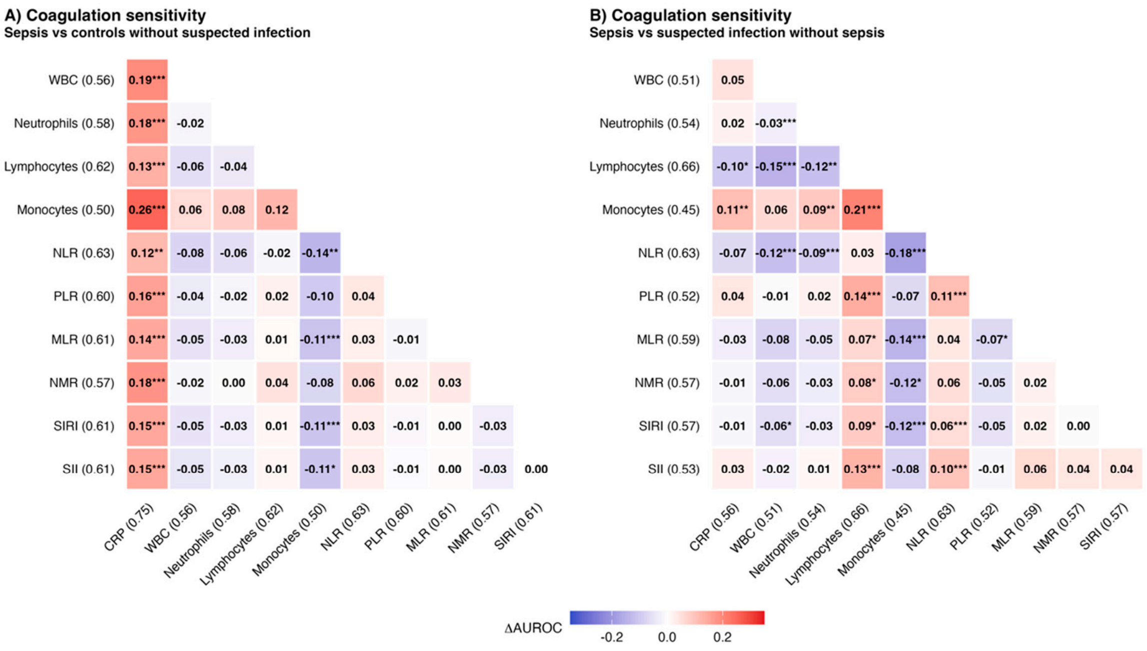

Panels A and B show the rematched sepsis versus controls without suspected infection and sepsis versus suspected infection without sepsis comparisons, respectively, after exclusion of sepsis episodes whose classification depended on points contributed by the coagulation component of SOFA. The analyses included 424 sepsis episodes and 166 controls without suspected infection (n=590) in Panel A and 424 sepsis episodes and 270 episodes with suspected infection without sepsis (n=694) in Panel B. AUROC values for each biomarker are shown in parentheses in the axis labels. Each cell represents ΔAUROC = AUROC of the column biomarker minus AUROC of the row biomarker; positive values indicate greater discrimination for the column biomarker and negative values greater discrimination for the row biomarker. Statistical significance was assessed using paired DeLong tests, with Holm adjustment across the 55 pairwise biomarker comparisons within each panel. *Holm adjusted $P<0.05$; **$P<0.01$; ***$P<0.001$. AUROC, area under the receiver operating characteristic curve; SOFA, Sequential Organ Failure Assessment.

Supplementary Figure S5. Nonlinear associations of absolute lymphocyte and neutrophil counts with sepsis in the matched comparison samples

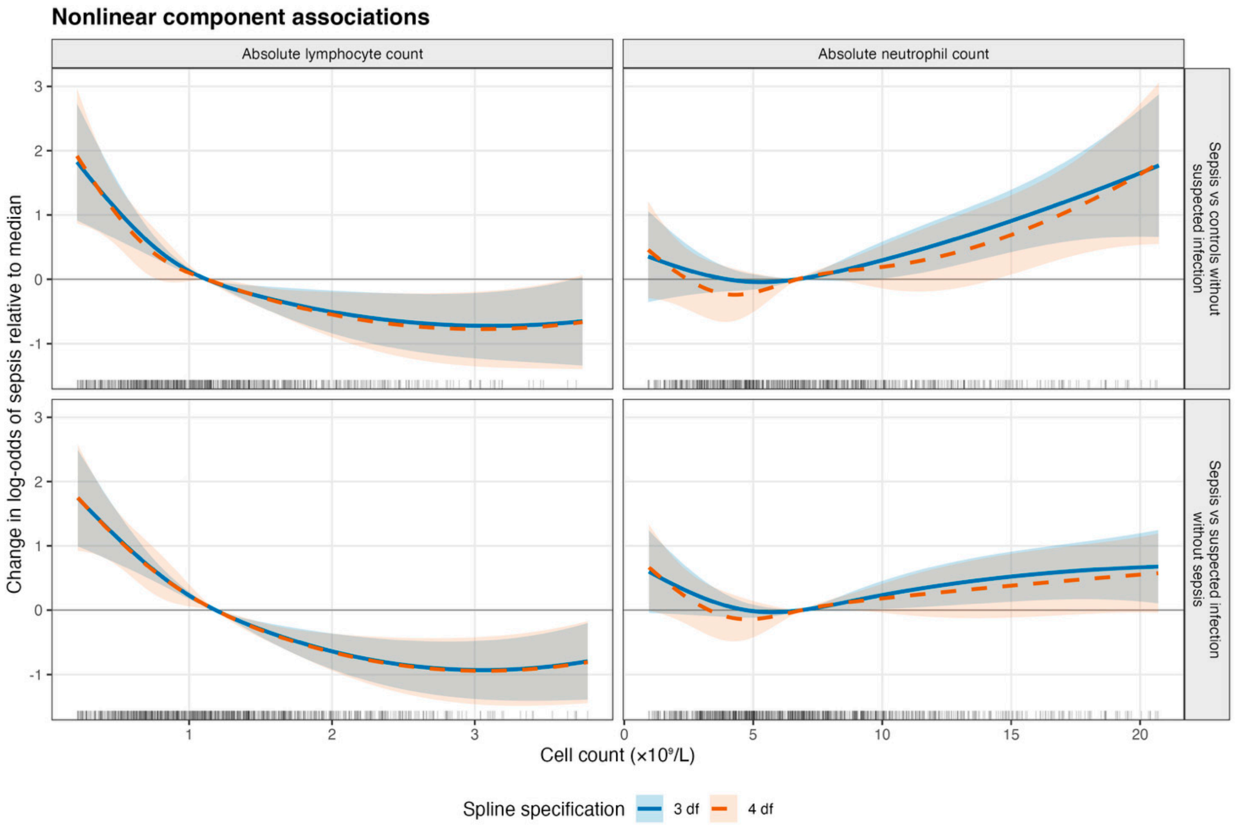

Curves were estimated from logistic regression models including both absolute lymphocyte and neutrophil counts, with each component modeled using natural cubic splines. The primary specification used 3 degrees of freedom (solid blue curves), and a 4 degree of freedom specification was examined as a sensitivity analysis (dashed vermillion curves). For each panel, the fitted association of the focal cell count with the log odds of sepsis was centered at its median value while the other cell count component was held at its sample median. Shaded areas indicate 95% Wald confidence intervals for the centered contrasts. Curves are displayed over the central 95% of observed values, and rug marks indicate the distribution of observations within the displayed range. The upper row compares sepsis with controls without suspected infection; the lower row compares sepsis with suspected infection without sepsis.
